# Effects of reciprocal transplantation on the microbiome and putative nitrogen cycling functions of the intertidal sponge, *Hymeniacidon heliophila*

**DOI:** 10.1038/srep43247

**Published:** 2017-02-24

**Authors:** Brooke L. Weigel, Patrick M. Erwin

**Affiliations:** 1 Department of Biology and Marine Biology, Center for Marine Science, University of North Carolina Wilmington, 5600 Marvin K. Moss Lane, Wilmington, NC 28409, USA

## Abstract

Microbial symbionts in sponges are ubiquitous, forming complex and highly diverse host-specific communities. Conspecific sponges display remarkable stability in their symbiont communities, both spatially and temporally, yet extreme fluctuations in environmental factors can cause shifts in host-symbiont associations. We previously demonstrated that the marine sponge *Hymeniacidon heliophila* displayed significant community-level differences in microbial symbiont diversity, structure and composition when sampled from intertidal and subtidal environments. Here, we conducted a 70-day reciprocal transplant experiment to directly test the effect of tidal exposure on the microbiome of *H. heliophila*, using next-generation Illumina sequencing of 16S rRNA gene sequences to characterize symbiont communities. While sponges transplanted between habitats displayed shifts in microbial communities after 70 days, temporal variation was the dominant factor affecting microbial community composition. Further, we identified core symbionts that persisted across these spatio-temporal scales and used a metagenomic approach to show that these dominant members of the microbiome of *H. heliophila* represent nitrogen cycling taxa that have the potential to contribute to a diverse array of nitrogen transformations in the sponge holobiont. Together, these results indicate that despite moderate spatio-temporal shifts in symbiont composition, core symbiont functions (e.g. nitrogen cycling) can be maintained in sponge microbiomes through functional redundancy.

Microorganisms influence many aspects of multicellular life, including the maintenance of genetic diversity through horizontal gene transfer, pathogenesis, provisioning of resources through nutrient cycling, and the acquisition of novel traits or resources via symbiotic associations^1^. Despite the widespread occurrence of animal-microbe associations among taxonomically diverse hosts and symbionts, the maintenance of these symbiotic associations across space and time is just beginning to be understood. Microbial symbionts in sponges (phylum Porifera) are ubiquitous, forming complex and host-specific communities that can contain thousands of unique microbial taxa^2^,^3^. Symbiotic microorganisms may benefit the sponge holobiont through photosynthesis^4^,^5^, nitrogen cycling^6^,^7^,^8^, hydrogen sulfide oxidation^9^, and the production of bioactive secondary metabolites^10^. Host specificity plays a major role in structuring symbiont communities, with many studies demonstrating substantial differences in microbial community structure and composition among different sponge species^11^,^12^,^13^,^14^. While interspecific variation in sponge-associated microbial communities is well established, relatively little is known about intraspecific variation in the sponge microbiome.

Spatial and temporal stability of sponge-microbe associations have been commonly reported. Bacterial symbionts of Mediterranean *Ircinia* spp. displayed temporal stability over 1.5 years^15^, spatial stability across distances of up to 800 km^16^, and resistance to change after experimental exposure to thermal stress and food shortage^17^. Studies using taxonomically diverse sponge species have demonstrated stable host-microbial symbiont communities over two^18^,^19^ and three successive years^14^. Despite evidence of widespread microbial symbiont stability in diverse sponge hosts, some conspecific sponge hosts have demonstrated temporal variation, including the Caribbean coral reef sponge *Axinella corrugata*^20^, and two sponges from the family Halichondriidae: *Halichondria panicea* from the North Sea^21^ and *Hymeniacidon sinapium* from the Yellow Sea^22^. Other studies have reported biogeographic variation in sponge-microbe associations^23^,^24^,^25^,^26^. A thorough understanding of how sponge symbiont communities vary in relation to their environment is an important prerequisite for addressing how sponge holobionts may be affected by, or acclimate to, changing environmental conditions in the future.

Changing environmental conditions may induce shifts in the community composition of symbionts associated with the host, which in turn can influence the function of the symbiotic interaction^27^. For example, nitrogen fertilization in grasslands can lead to less mutualistic mycorrhizal fungi strains that exert a higher net carbon cost on their host^28^,^29^. In corals, thermal stress can alter host-symbiont interactions by causing bleaching, or the expulsion of symbionts, followed by a change in the composition of *Symbiodinium* clades that recolonize the coral after the bleaching event (i.e. symbiont shuffling^30^). In sponges, tissue necrosis and shifts in the relative abundance of microbial taxa have been shown to follow disturbances, including experimental warming^31^, host disease^32^,^33^, and exposure to heavy metals^34^. Relatively few studies have examined how environmental factors may disrupt the stability of symbiont communities in sponges, an important aspect of sponge biology as marine environments are exposed to further anthropogenic disturbances related to climate change^35^. Because variability in the sponge microbiome reflects a response of the host sponge and its microbial symbionts to changes in a multitude of ecological factors, conducting manipulative studies will help to elucidate the key factors that alter the stability of host-symbiont associations.

Characterizing the microbiome of sponges before and after experimental transplantation between contrasting environments can provide empirical insight into the environmental shaping of the sponge microbiome. Such transplantation experiments in sponges are scarce and have demonstrated a high degree of microbial symbiont stability despite transplantation between different habitats. For example, reciprocal transplantation between high and low-light habitats did not affect the overall microbial symbiont communities of *Tethya bergquistae* and *Ecionemia alata* after six weeks^36^. Transplanting the sponge *Petrosia ficiformis* from an illuminated site to a dark cave had no overall effect on the microbial symbiont community after five months, with the exception of a greater relative abundance of the ammonia-oxidizing archaeon *Nitrosopumilus* sp. in the sponge that was transplanted into the cave^25^. In another study, *Aplysina cavernicola* transplanted from deep (40 m) to shallow, more illuminated sites (<15 m) for three months displayed very similar microbial symbiont communities and identical natural product chemical profiles before and after transplantation^37^. Another study transplanted coral reef sponges (*Aplysina archeri* and *Desmapsamma anchorata*) to mangrove roots for 50 and 29 days, respectively, and found no differences in microbial symbiont communities^38^. Thus far, transplantation experiments have demonstrated that microbial symbiont communities in sponges are remarkably resistant to experimentally induced environmental change.

Conspecific sponges display remarkable stability in their symbiont communities, yet extreme fluctuations in environmental factors can cause shifts in host-symbiont associations. We previously demonstrated that the marine sponge *Hymeniacidon heliophila* displayed significant differences in microbial symbiont community diversity, structure and composition when sampled from intertidal and subtidal environments^39^. Differences in the microbial symbiont communities of intertidal and subtidal conspecific *H. heliophila* included shifts in the relative abundance of dominant taxa as well as a unique composition of rare symbionts in each environment. Interestingly, one of the dominant microbial OTUs that displayed different relative abundances in intertidal and subtidal hosts was the alphaproteobacterium *Thalassobaculum litoreum*^39^, which is a nitrate reducing species^40^. Given these differences in the structure, diversity and composition of intertidal and subtidal sponge microbiomes, with possible differences in nitrogen cycling functions^39^, we decided to further investigate nitrogen cycling in *H. heliophila*. Archaeal ammonia oxidizing genes (*amoA*) have been reported in the *H. heliophila* microbiome^41^, but microbial nitrogen cycling functions have never been holistically examined in an intertidal sponge species.

In this study, we conducted a reciprocal transplant experiment to directly test the effects of tidal exposure on the microbial symbiont community of *H. heliophila*, which is amenable to transplantation within the littoral zone. First, we addressed whether reciprocal transplantation of *H. heliophila* between intertidal and subtidal environments can induce changes in microbial symbiont community structure and composition. To address this question, we characterized the microbial symbiont community of *H. heliophila* before and after 70 days of experimental transplantation using next-generation Illumina sequencing of bacterial/archaeal 16S rRNA gene sequences. Surprisingly, temporal variation during the reciprocal transplant experiment accounted for most of the variation in microbial symbiont community structure, thus we analyzed this dataset alongside previously collected 16S rRNA microbial community data from *H. heliophila* to compare monthly and annual variation in the microbial symbiont communities. Finally, we addressed whether changes in the microbial community structure of *H. heliophila* were accompanied by changes in the functional community of nitrogen cycling symbionts. To address this objective, we identified key nitrogen cycling processes in microbial symbionts of *H. heliophila* using a predictive metagenomic approach for all sponge individuals and verified these predictions by comparison with nitrogen cycling genes identified in a shotgun-sequenced *H. heliophila* metagenome for one sponge individual.

## Results

### Transplant survival and temperatures regimes

**Figure 1:**
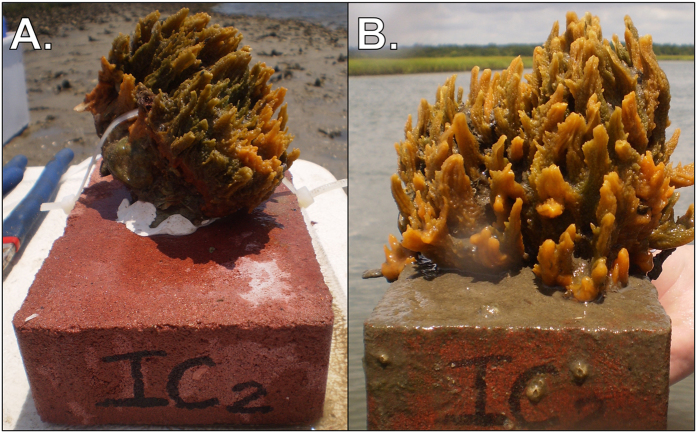
*H. heliophila* intertidal control (replicate #2) in May 2015 (**A**), and after 70 days of intertidal exposure in July (**B**).

After 70 days of reciprocal transplantation, the sponges appeared healthy and many had visually increased in size from May to July 2015 (Fig. 1). While sponge mortality was not directly observed, 11 experimental bricks were recovered without sponge tissue. Sponges may have died and separated from the bricks, but it is also possible that physical disturbance caused their disappearance. Notably, the proportion of surviving sponges was equal in intertidal-control and intertidal-to-subtidal treatments (62.5%), lower in the subtidal-control (50%), and drastically lower in the subtidal-to-intertidal treatment (25%). Temperature loggers revealed large differences in the daily range of temperatures between intertidal and subtidal environments over the duration of the experiment; the intertidal environment experienced an average daily range more than twice that of the subtidal (10.1 °C ± 4.4 and 4.7 °C ± 1.3, respectively; Supplementary Fig. S1). Further, temperature extremes during the 70-day experimental period were much greater in the intertidal (maximum, 44.5 °C; minimum, 17.3 °C) than the subtidal (maximum, 34.1 °C; minimum, 22.5 °C). However, the average temperatures from intertidal and subtidal environments were similar (27.9 °C ± 2.7 and 27.5 °C ± 2.0). Temperatures increased over time, but the magnitude of increase was similar in intertidal and subtidal environments; temperatures increased by 1.83 °C and 2.11 °C, respectively, from May to June 2015, and by 0.20 °C and 0.17 °C, respectively, from June to July 2015.

### Microbial symbiont community composition and diversity

**Figure 2:**
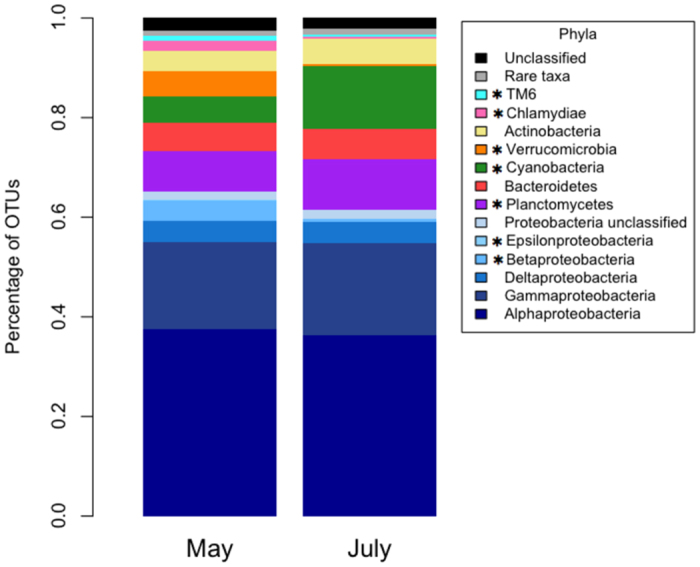
Phylum level microbial community composition of pooled replicates in May (*n* = 16) and July 2015 (*n* = 16), with Proteobacteria further divided by class. Asterisks (*) indicate significant differences of phyla between May and July (paired t-tests; d.f. = 15, *P* < 0.05). Rare taxa include (in order of decreasing abundance): Acidobacteria, *OP11, Chloroflexi, Parvarchaeota, *WS5, Crenarchaeota, OD1, Gemmatimonadetes, Firmicutes, TM7, OP3, WS3, Chlorobi, Fusobacteria, Spirochaetes, Euryarchaeota, Lentisphaerae, BRC1, SBR1093, Tenericutes, WPS-2, Caldithrix, Fibrobacteres, KSB3, LD1, OP8, PAUC34f, SAR406, Thermi, Elusimicrobia, GN02, NKB19, SR1, VHS-B3–43, WS2, WWE1, and ZB3.

In total, *H. heliophila* samples from May and July 2015 yielded 7,722 OTUs that belonged to 56 bacterial phyla and 3 archaeal phyla (*Crenarchaeota, Euryarchaeota*, and *Parvarchaeota*). The bacterial symbiont community of *H. heliophila* was dominated by *Proteobacteria* (63%), which were mostly composed of *Alphaproteobacteria* (37% of total symbiont community) and *Gammaproteobacteria* (18%). Other abundant phyla included *Planctomycetes* (9.2%), *Cyanobacteria* (8.9%), *Bacteroidetes* (5.9%), and *Actinobacteria* (4.5%). Despite experimental transplantation between intertidal and subtidal environments, the largest differences in microbial symbiont community composition among all treatment and control sponges occurred between the start (May 2015) and end (July 2015) of the experiment (Fig. 2). Many phyla displayed significantly different abundances in May and July, including relatively abundant phyla (*Betaproteobacteri*a, *Cyanobacteria, Planctomycetes*, and *Verrucomicrobia*) as well as less abundant phyla (*Epsilonproteobacteria, Chlamydiae, TM6, OP11, WS5*; paired t-tests; d.f. =15, *P* < 0.05; Fig. 2). At the OTU level, there were 282 taxa that had significantly different relative abundances in May and July (Metastats, *P* < 0.05, Supplementary Table S1). There were ten OTUs that contributed a cumulative 22% to the observed dissimilarity between May and July (as revealed by SIMPER) and had significantly different relative abundances (Metastats, *P* < 0.05; Supplementary Fig. S2; Supplementary Table S1).

Total OTU richness was higher in May (*n* = 4,878) than July 2015 (*n* = 3,985). There was a significant effect of month on mean OTU richness (*S*) and Shannon diversity (*H’*), both of which were significantly higher in May than July 2015 (two-way ANOVA; *F* = 10.16, d.f.=1, *P* = 0.004 for *S*; *F* = 4.69, d.f.=1, *P* = 0.041 for *H’*; Table 1). The inverse Simpson index (*D*) was also higher in May, though this difference was not significant (two-way ANOVA; *F* = 2.70, d.f = 1, *P* = 0.113; Table 1). While all three diversity indices were highest in the subtidal-control and subtidal-to-intertidal treatments, there was no significant effect of treatment on OTU richness (two-way ANOVA; *F* = 2.47, d.f. = 3, *P* = 0.086), Shannon diversity (two-way ANOVA; *F* = 0.882, d.f. = 3, *P* = 0.464), or the inverse Simpson index (two-way ANOVA; *F* = 0.53, d.f. = 3, *P* = 0.666; Table 1). Finally, the interaction between month and treatment did not have a significant effect on alpha diversity indices (two-way ANOVA; *F* = 1.8, d.f. = 3, *P* = 0.173 for *S*; *F* = 0.87, d.f. = 3, *P* = 0.47 for *H’*; *F* = 1.72, d.f. = 3, *P* = 0.191 for *D*).

### Microbial symbiont community structure

**Figure 3:**
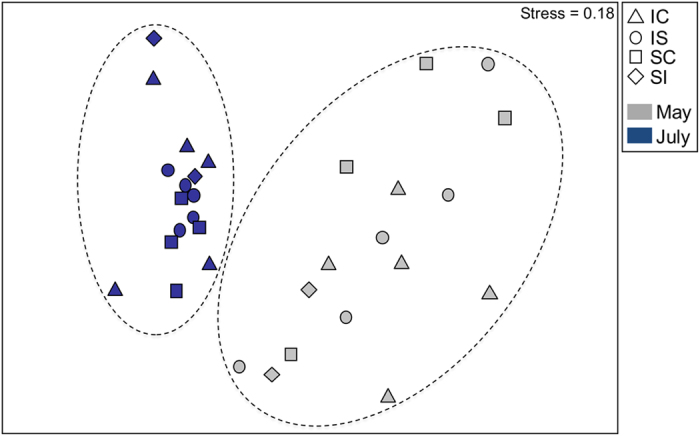
nMDS plot showing significantly different microbial symbiont community structures between *H. heliophila* from May and July 2015. Dashed lines indicate the groupings of sponge samples from May (grey) and July (blue). Symbols denote different transplant treatments: intertidal-control (triangles), intertidal-to-subtidal (circles), subtidal-control (squares), subtidal-to-intertidal (diamonds).

Variation in the microbial symbiont community structure of *H. heliophila* was primarily driven by the sampling month (PERMANOVA; *Pseudo-F* = 8.06, d.f. = 1, *P* = 0.001), which explained 32% of the variability in microbial community structure. Treatment and month*treatment were not significant factors (PERMANOVA; *Pseudo-F* = 1.16, d.f. = 3, *P* = 0.186 for treatment; *Pseudo-F* = 1.19, d.f. = 3, *P* = 0.199 for month*treatment). Finally, the sponge individual (nested in treatment) factor was not significant (PERMANOVA; *Pseudo-F* = 1.04, d.f. = 12, *P* = 0.397), indicating that the sponge individuals did not significantly contribute to the variation in microbial symbiont community structure. The permutational multivariate analysis of dispersion was significant for the factor of month (PERMDISP; *F* = 16, *P* = 0.002), indicating unequal variances between May and July. These trends are visible in the nMDS plot, as microbial community samples were more tightly clustered in July compared to May, yet clusters from the two months were non-overlapping (Fig. 3). The average similarity in microbial communities between sponges sampled in May and July 2015 was 46%.

Pairwise comparisons revealed no structural differences among the treatments in May while significant pairwise differences were observed between some experimental treatments in July (PERMANOVA pairwise tests; Table 2). In July, the subtidal-control and subtidal-to-intertidal treatments exhibited a trend toward significance (*P* = 0.058), while the intertidal-control differed significantly from subtidal-control and intertidal-to-subtidal treatments (Table 2). Further, the intertidal-control treatment did not differ from the sponges that were transplanted to the intertidal (i.e., subtidal-to-intertidal treatment), and the subtidal-control was not different from the sponges that were transplanted to the subtidal (i.e., intertidal-to-subtidal treatment). Notably, the subtidal-to-intertidal and intertidal-to-subtidal treatments did not display significantly different microbial community structures in July (Table 2); however, the low sample size of the subtidal-to-intertidal treatment (*n* = 2) likely affected the statistical power of pairwise comparisons that included this treatment and could lead to a type II error.

While the differences between treatments were less pronounced, there were still detectable differences in OTU abundances between treatments. In July, there were 26 OTUs that had significantly different relative abundances between intertidal-control and subtidal-control treatments (Metastats, *P* < 0.05, Supplementary Table S2), contributing to a cumulative 15.4% of the dissimilarity between these treatments (as revealed by SIMPER). There were 42 differentially abundant OTUs between intertidal-control and intertidal-to-subtidal treatments (Metastats, *P* < 0.05, Supplementary Table S2), contributing to 14.5% of the dissimilarity between these treatments. Notably, the most abundant OTU1, the alphaproteobacterium *Novosphingobium resinovorum*, contributed between 8–11% to the dissimilarity between treatments and exhibited a greater mean abundance in the subtidal-control and intertidal-to-subtidal treatments (Metastats, *P* < 0.05). OTU12, an alphaproteobacterium in the family Rhodobacteraceae, contributed to roughly 1.2% of the dissimilarity between treatments and was significantly more abundant in the intertidal-control and subtidal-to-intertidal treatments (Metastats, *P* < 0.05).

### Annual and monthly comparison of *H. heliophila* microbial communities

Collective analyses of *H. heliophila* microbial communities from 2014 and 2015 revealed significant effects of both month and year on microbial community structure (PERMANOVA; *Pseudo-F* = 9.21, d.f. = 1, *P* = 0.001 for month; *Pseudo-F* = 3.14, d.f. = 1, *P* = 0.001 for year). A stronger effect of month (accounting for 31% of total variation) than year (9%) was observed on *H. heliophila* microbial symbiont community structure. Accordingly, May 2015 sponges exhibited distinct community structures from July (2014 and 2015) sponges (Supplementary Fig. S3). July 2014 and 2015 samples exhibited a high degree of similarity, yet July 2014 samples collected from intertidal sponges formed a distinct cluster within all of the July samples (Supplementary Fig. S3). At the OTU level, May 2015 sponges had higher total species richness (*n* = 4,878), while July 2014 (*n* = 3,745) and July 2015 (*n* = 3,985) samples exhibited similar species richness.

### Predicted and actual nitrogen cycling functional genes in *H. heliophila*

**Figure 4:**
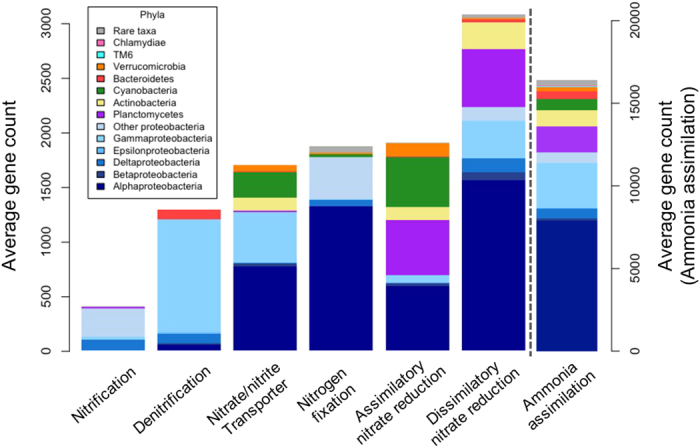
PICRUSt predicted average gene counts and phylum-level taxa associated with each nitrogen transformation pathway in *H. heliophila*. Note that ammonia assimilation has a secondary axis to account for the order of magnitude difference in ammonia assimilation genes compared to other nitrogen transformations. Rare taxa include (in order of decreasing abundance): Parvarchaeota, Acidobacteria, Gemmatimonadetes, OD1, Spirochaetes, Chloroflexi, Firmicutes, OP11, TM7, Euryarchaeota, WS5, OP3, SAR406, BRC1, NKB19, PAUC34f, GN02, SBR1093, Fusobacteria, WS3, Crenarchaeota, Caldithrix, LD1, KSB3, Fibrobacteres, Thermi, OP8, Lentisphaerae, Chlorobi, WS2, and WPS-2.

A comparison of the shotgun sequenced metagenome and the PICRUSt predicted metagenome from the same *H. heliophila* sample revealed similar nitrogen cycling functional genes (Supplementary Fig. S4), with 25 nitrogen metabolism KOs in the predicted metagenome and 22 in the actual metagenome. Of these, 12 nitrogen metabolism KOs spanning a diverse range of functional pathways (ammonia assimilation, ammonia oxidation, assimilatory nitrate reduction, dissimilatory nitrate reduction, and denitrification) were found in both the predicted and actual metagenome (Supplementary Table S3). Ammonia assimilation was the dominant nitrogen cycling functional pathway, accounting for 62% of predicted and 78% of actual nitrogen metabolism genes (Supplementary Fig. S4). The next largest functional group included denitrification and dissimilatory nitrate reduction genes, combined comprising 16% of predicted and 14% of actual nitrogen metabolism genes. Ammonia oxidation genes accounted for only 2% of the predicted and 3% of the actual nitrogen cycling metagenome. Notably, in the actual metagenome, there were genes for all steps of the denitrification pathway including both membrane-bound (*narB, narZ*) and periplasmic (*napA, napC*) nitrate reductases, nitrite reductases (*nirA, nirB*), nitric oxide reductases (*norB, norC, norF*), and nitrous oxide reductase (*nosZ*). We predicted the phylum-specific gene counts for key nitrogen cycling processes in all *H. heliophila* samples. *Proteobacteria* was the only phylum that contributed to all nitrogen transformation processes and was also the dominant phylum within each process (Fig. 4). Other major contributors to nitrogen cycling genes included the phyla *Planctomycetes, Actinobacteria, Cyanobacteria, Bacteroidetes* and *Verrucomicrobia* (Fig. 4).

### Core microbial symbiont diversity and putative functionality

**Figure 5:**
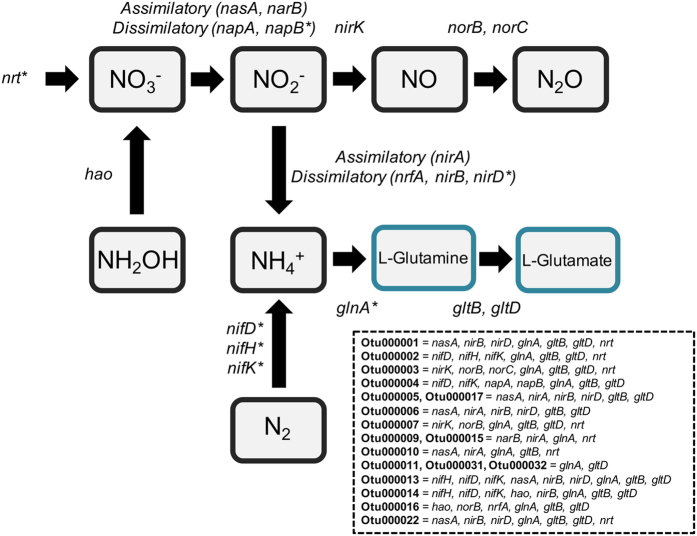
Nitrogen transformation pathways and associated genes predicted in *H. heliophila* core microbial symbionts using PICRUSt. Asterisks (*) indicate genes predicted to exist in the core microbial community that were not present under the KEGG gene annotation system in the single shotgun sequenced metagenome.

The total OTU richness from all *H. heliophila* samples was 10,410, yet the core microbial community (defined here as OTUs detected in all 44 samples) consisted of only 18 OTUs (Table 3). Many of the core OTUs were the most abundant taxa and the core symbiont community accounted for the majority (63%) of the total microbial community of *H. heliophila* (range within individual sponges = 45–79%). Core microbial symbionts included *Proteobacteria (n* = 8), *Bacteroidetes (n* = 3), *Planctomycetes (n* = 2), *Cyanobacteria (n* = 2), *Actinobacteria (n* = 1), *Verrucomicrobia (n* = 1), and *Spirochaetes (n* = 1). All 18 of the core OTUs displayed putative nitrogen cycling functions in their predicted genomes. Together, the core symbiont community exhibited putative nitrogen metabolism genes involved in ammonia assimilation, nitrogen fixation, ammonia oxidation (hydroxylamine dehydrogenase), assimilatory and dissimilatory nitrate/nitrite reduction, and denitrification (Fig. 5).

### Temporal trends in nitrogen cycling functional communities

Despite the change in microbial community composition between sponges sampled in May and July 2015, there was no difference in total predicted nitrogen cycling functional gene counts over time (paired t-test; *t* = −1.91, d.f. =15, *P* = 0.08). The only nitrogen cycling pathway that differed significantly between May and July was assimilatory nitrate reduction, which was higher in July (paired t-test; *t* = −2.39, d.f. = 15, *P* = 0.03). Ammonia assimilation, ammonia oxidation, nitrogen fixation, dissimilatory nitrate reduction, and denitrification gene counts did not display significant temporal variation (paired t-tests; d.f. =15, *P* > 0.05).

## Discussion

Previously, we demonstrated variation in the microbiome of *H. heliophila* from intertidal and subtidal environments, with significant differences in the composition of both dominant and rare members of the microbiome^39^. Herein, a reciprocal transplant experiment was conducted between the same intertidal-subtidal environments and demonstrated that transplantation of sponges between habitats induced shifts in their microbial communities. These treatment differences were driven by shifts in a few microbial taxa, including a greater abundance of the most common OTU1 (alphaproteobacterium, *Novosphingobium resinovorum*) in subtidal treatments and a greater abundance of OTU12 (alphaproteobacterium, family Rhodobacteraceae) in intertidal treatments. While some inter-annual variability in the magnitude and assortment of compositional differences was detected between intertidal and subtidal sponges (July 2014^39^ vs. July 2015, this study), the shifts in dominant symbiont taxa that we observed in experimental sponges indicate that tidal exposure does select for a unique consortium of microbial taxa in each environment.

This study also revealed that the microbiome of *H. heliophila* displays temporal variation, with significantly different microbial symbiont community structure, composition, and diversity between sponges sampled in May and July 2015. In fact, temporal variation was the dominant factor affecting microbial community composition in the reciprocal transplant experiment. To date, few sponge species have been reported to demonstrate temporal variability in symbiont structure^14^ and most represent low microbial abundance (LMA) sponges^20^,^21^,^22^, with the exception of the high microbial abundance (HMA) sponge *Aplysina cauliformis*^33^. Similarly, recent studies in the Mediterranean Sea reported species-specific bacteria in both sponge groups^42^, yet greater temporal variability of microbial communities associated with LMA than HMA sponge hosts over one year^43^. Our results support the hypothesis that some LMA sponges display significant temporal variability, as *H. heliophila* has been classified as a LMA sponge based on transmission electron microscopy observations and microbial species composition^44^,^45^. Interestingly, two sponge species for which temporal variation has been previously reported are closely related to *H. heliophila*: the congeneric sponge *H. sinapium*^22^ and *Halichondria panacea*^21^, which belong to the same family (Halichondridae) as *H. heliophila*. Further, spatial variation in archaeal communities of *H. heliophila* was detected between sponges collected inside and outside of a polluted estuary in Brazil^41^. Together, these results suggest that certain taxonomic groups of LMA sponges exhibit more dynamic microbial symbiont communities than HMA sponges, thus representing important targets for understanding how host-symbiont interactions vary in response to changing environmental conditions.

Temporal variation in the sponge microbiome may result from a physiological response of the microbial community to temporal fluctuations in abiotic factors, from differentially adaptive microbial symbionts during life history-associated seasonal changes in the sponge host, or a combination of both effects. While temperature represents an important driver of seasonal variation in free-living microbial communities^46^, greater differences in temperature regimes did not account for greater variation in the microbiome of *H. heliophila*. Rather, we report greater variation in sponge microbiomes over time (coinciding with a modest temperature increase) than between intertidal and subtidal habitats (where markedly different temperature regimes occurred), indicating that temperature may not play a major role in driving temporal variation in the microbiome of *H. heliophila*. Other environmental factors that could have affected the observed temporal variation include salinity, dissolved oxygen levels, or the availability of food resources to the sponge, but these variables were not measured in this experiment. Temporal variation in symbiont communities of *H. heliophila* may also be linked to seasonal cycles in host sponge reproductive processes, such as embryogenesis and gametogenesis, as reported for the congeneric intertidal sponge *H. sinapium* from the Yellow Sea^22^. *H. sinapium* exhibited clear seasonal variation in microbial symbiont communities over 1.5 years that coincided with four distinct developmental and reproductive stages of the host sponge^22^. Future studies incorporating host life cycle data alongside other temporally variable environmental factors are needed to further elucidate the drivers of variation in the sponge microbiome.

Our study also revealed insight into the interactive effects of season and habitat on microbial symbiont community structure. During the summer season (July), significant differences were detected between intertidal and subtidal sponge microbial communities, consistent with previous results^39^; however, these differences were not detected during the spring season (May). We hypothesize that annual re-colonization of intertidal habitat by subtidal sponge individuals may explain the lack of microbial community variation between intertidal and subtidal sponges in May. Intertidal populations of *H. heliophila* decrease markedly during the winter months in North Carolina^47^ (B. Weigel pers. obs.), possibly due to exposure to low air temperatures as this is the northern limit for the intertidal form of *H. heliophila*^47^. As spring approaches, intertidal populations of *H. heliophila* increase in North Carolina and may include colonization by overwintering, subtidal sponges. While more detailed observations of the seasonal dynamics of *H. heliophila* are necessary, the greater differences between intertidal and subtidal hosts in summer compared to spring may reflect adaptation of microbial communities to the intertidal environment throughout the summer.

In addition to investigating variation in microbial symbiont community structure, we examined functional genes involved in nitrogen cycling and how predicted gene counts varied over time. Nitrogen metabolism genes identified in both the predicted and actual metagenomes of *H. heliophila* represented a broad functional diversity of nitrogen transformations, including ammonia assimilation, ammonia oxidation, assimilatory nitrate reduction, dissimilatory nitrate reduction, and denitrification. Ammonia assimilation was the dominant nitrogen transformation pathway in the *H. heliophila* holobiont (ca. 60–80% of all predicted and actual nitrogen metabolism genes), consistent with studies showing very high rates of ammonium excretion by sponges^48^,^49^. While some sponge species host diverse ammonia-oxidizing symbionts^32^,^50^, ammonia oxidation genes accounted for only 2–3% of nitrogen metabolism genes in *H. heliophila*. Genes involved in denitrification and dissimilatory nitrate reduction pathways were detected, accounting for ca. 15% of all nitrogen metabolism genes. Denitrification has been quantified in multiple sponge species^6^,^7^ and genes for nitrite (*nirK*) and nitrous oxide reductase (*nosZ*) have been detected in diverse sponge species^51^. The presence of the complete suite of denitrification genes in the *H. heliophila* holobiont suggests that nitrate can be reduced all the way to atmospheric N_2_ gas. Nitrogen transformations were predicted to occur primarily in the bacterial phyla *Proteobacteria, Planctomycetes, Actinobacteria, Cyanobacteria, Bacteroidetes* and *Verrucomicrobia*. The core microbiome of *H. heliophila* consisted of 18 OTUs, which together comprise an average of 63% of the total microbial community of *H. heliophila*, and each member of the core microbiome was predicted to be associated with at least one nitrogen metabolism gene. These results suggest that nitrogen cycling taxa are dominant, persistent members of the microbiome of *H. heliophila* and may perform nitrogen transformations of ecological importance to the sponge host.

Determining the extent of functional redundancy in microbial symbiont communities is a current research priority in the field of sponge microbiology, as this knowledge aids in understanding and predicting how the sponge holobiont will respond to changing environmental conditions^52^. While the symbiont community composition in *H. heliophila* displayed significant temporal variation, no corresponding differences in predicted functional gene counts were detected, suggesting that core nitrogen cycling processes were maintained through functional redundancy. The sole exception was a significant difference in assimilatory nitrate reduction genes over time; however, these genes comprised a small proportion of all nitrogen cycling genes in *H. heliophila* (Supplementary Fig. S4). Previous work suggests that specific functions of the sponge microbiome are conserved across different host species, as the Mediterranean sponges *Agelas oroides* and *Chondrosia reniformis* harbored distinct microbial communities but displayed similar rates of nitrification and nutrient uptake^53^. Additionally, functionally similar nitrogen cycling genes were detected in six taxonomically diverse sponge species with divergent microbial communities^54^. Functional redundancy is likely to play a role in microbial communities with high genetic diversity, as nutrient cycling functions can be performed by diverse taxa and some microbial groups display flexibility in their metabolic functions^55^. Furthermore, selective pressures may maintain nutrient cycling functionality in sponge-microbe symbioses if these metabolic processes are important for host sponge physiology (e.g. nitrogenous waste removal). While we report stable nitrogen cycling functions in the microbiome of *H. heliophila* following temporal shifts in community composition and seasonal changes in temperature, exceeding thermal stress limits can cause drastic changes in microbial community structure and functional collapse of the sponge holobiont^56^. Future work should seek to understand how the functionality of the sponge holobiont is affected by temporal variation in microbial community structure, as well as how more extreme environmental perturbations and future climate regimes may impact the stability and health of the sponge holobiont.

## Methods

### Reciprocal transplant experimental design

The study site was located within Loosin Creek (34.1722N, −77.8328W) in Wilmington, North Carolina. Loosin Creek has a salinity range of 22–35 ppt and a tidal range that averages 1.2 meters. The benthic habitat is composed of soft mud, sand and patches of oyster reef. On 18 May 2015, individuals of the marine sponge *Hymeniacidon heliophila* (Parker 1910) were collected during a low spring tide from intertidal (completely exposed to air) and subtidal (1–2 m below low tide) environments. Sponge individuals from this collection site were previously genotyped using ITS-2 and partial 28S rDNA sequences, and intertidal and subtidal sponges exhibited identical genotypes^39^. Eight subtidal *H. heliophila* were collected and divided into halves of approximately equal size. For each subtidal sponge, one half was transplanted from the subtidal to the intertidal (SI) and one half was returned to the subtidal (subtidal-control; SC). Likewise, eight intertidal *H. heliophila* were collected and divided in half: one half of each sponge was transplanted from the intertidal to the subtidal (IS), and the other half was returned to the intertidal (intertidal-control; IC). All sponges were attached to labeled bricks in the field using cable ties and underwater epoxy. HOBO temperature loggers were attached to one intertidal and one subtidal sponge, and temperatures were recorded at 15-minute intervals. All specimens of *H. heliophila* were photographed and sampled for microbial community characterization before and after 70 days, on 18 May and 27 July 2015. Of the 32 experimental sponge bricks, five replicates were not recovered at the end of the experiment (1 IC, 2 SC and 2 IS) and eleven were recovered without sponges (2 IC, 2 SC, 1 IS and 6 SI). After 70 days of reciprocal transplantation, the 16 sponge individuals that we were able to recover were sampled (final *n* for each treatment and control group: IC = 5, SC = 4, IS = 5, SI = 2; see Supplementary Table S4). Sponge tissue samples collected on each date were immediately stored in 95% ethanol. Ambient seawater samples were not collected for this experiment, although past studies revealed low overlap and clear differentiation between the microbiome of *H. heliophila* and environmental communities in sediment and seawater^39^,^45^. DNA was extracted from sponge tissue samples that were paired between May and July (*n* = 32 total) using the DNeasy^®^ Blood & Tissue Kit (Qiagen). An additional twelve *H. heliophila* samples, previously collected from subtidal (*n* = 6) and intertidal (*n* = 6) environments in July 2014 and treated with the same DNA extraction and next-generation sequencing and processing protocols^39^, were included for comparative analyses of inter-annual variation in microbial community structure and diversity.

### Next generation DNA sequence processing and statistical analysis

DNA extracts were sent to Molecular Research LP (Shallowater, TX) for amplification, library construction and multiplexed sequencing of partial (V4) 16S rRNA gene sequences using the universal bacterial/archaeal forward primer 515f and reverse primer 806r^57^ on an Illumina MiSeq platform. Raw sequences were processed using a modified version of the Illumina MiSeq SOP pipeline^58^ (http://www.mothur.org/wiki/MiSeq_SOP) in mothur, as described in Weigel and Erwin^39^. Briefly, raw sequence reads were quality-filtered, aligned to the Greengenes reference database (gg_13_5_99) and trimmed to the V4 region, screened for sequencing errors, and taxonomically classified. Non-target taxa (chloroplasts, mitochondria and eukarya) were removed from the dataset and high quality sequences were assigned to operational taxonomic units (OTUs) in mothur, using 97% sequence identity and the average neighbor clustering algorithm. Due to varied sampling depths (i.e. number of sequence reads) among replicates, each dataset was subsampled to the lowest read count (*n* = 2,853) from the final shared file (Supplementary Table S4). All subsequent data analyses were based on the final subsampled datasets. Raw sequence data were deposited as FASTQ files in the Sequence Read Archive of the National Center for Biotechnology Information (SRA NCBI) under the accession no. SRP076523. Previously published raw sequences from intertidal and subtidal sponges collected in July 2014 can be found in the NCBI Sequence Read Archive under the accession number SRP065064^39^ (Supplementary Table S4).

To compare microbial community structure among the treatments and between months, Bray-Curtis similarity matrices were created using OTU abundances in PRIMER (version 6.1.11). Bray-Curtis similarity matrices were visualized using nonmetric multidimensional scaling (nMDS) plots and cluster dendrograms. A three-way factorial permutational multivariate analysis of variance (PERMANOVA) was used to test for significant differences in microbial community structure between May and July 2015 and among the treatments by including the fixed factors ‘month’ and ‘treatment’, and their interaction term ‘month*treatment’. The random factor ‘sponge individual (nested in treatment)’ was included in the PERMAOVA design to account for possible individual sponge-specific effects due to resampling the same sponges in May and July. To test for significant differences among all *H. heliophila* microbial communities from July 2014, May 2015 and July 2015, a PERMANOVA was used with month and year as fixed factors. PERMANOVA pairwise comparisons were conducted for all factors, including one case when the main test was not significant, since the nature of main tests (by considering both pairwise and non-pairwise comparisons) can mask significant pairwise tests^59^. Multiple pairwise comparisons were corrected based on the Benjamini-Yekutieli false discovery rate control and an experiment-wise error rate of α = 0.05. To test for unequal dispersion of variability among groups, permutational multivariate analyses of dispersion (PERMDISP) were conducted for all significant PERMANOVA outcomes.

To detect microbial taxa that contributed to the dissimilarity between microbial communities in May and July 2015, and among significantly different treatments in July 2015, we used one-way similarity percentages species contributions (SIMPER) analyses with OTU abundances in PRIMER. In order to detect differentially abundant OTUs, Metastats^60^ was run in Mothur with 1,000 permutations to compute non-parametric *P* values based on two-sample *t*-tests. After detecting significantly different OTUs using Metastats, we used SIMPER to quantify the contribution of differentially abundant OTUs to the overall dissimilarity between groups. To identify microbial phyla that differed significantly in abundance between May and July 2015, paired two-sided Student’s t-tests were run in R using phylum-level abundances.

To compare microbial community diversity among the treatments, the alpha diversity indices OTU richness (*S*), Shannon–Weaver diversity (*H’*) and the inverse Simpson’s index (*D*) were calculated using the R package vegan 2.0–10^61^ and R script written by Easson and Thacker^13^. To examine the effects of treatment and month on the alpha diversity indices, two-way analyses of variance (ANOVA) were run in R with factors of ‘month’, ‘treatment’, and their interaction term ‘month*treatment.’

### Predictive metagenomic analysis

To predict the metagenome of each sponge microbial community sample and infer putative functionality, the bioinformatics program PICRUSt^62^ was used. PICRUSt uses 16S rRNA data to predict the metagenome of each sample by matching previously sequenced reference genomes to each OTU from the dataset. Specifically, OTU abundances from the final subsampled dataset were used with the predict_metagenomes.py script in Python to generate collective metagenome predictions for each sample based on the Kyoto Encyclopedia of Genes and Genomes (KEGG) orthology (KO) database. In order to assess the accuracy of the predicted metagenomes, a weighted Nearest Sequenced Taxon Index (NSTI) score was generated for each sample. NSTI scores are a measure of the phylogenetic distance between each empirical OTU and their reference genome match. The accuracy of PICRUSt decreases with increasing NSTI scores, but scores ≤ 0.17 produced accurate metagenome predictions^62^. The mean (±standard deviation) NSTI score for all *H. heliophila* predicted metagenomes from the 2015 transplant experiment was 0.105 (±0.01). Finally, to obtain OTU-specific gene counts for key nitrogen cycling processes in the metagenome, the metagenome_contributions.py script with the –l option was used for the following KEGG orthologs: nitrogen fixation (K00531, K02586, K02588, K02591), ammonia oxidation (K10535, K10944, K10945, K10946), denitrification (K00368, K00376, K04561, K02305, K15864,), dissimilatory nitrate reduction (K00370*, K00371*, K00373*, K00374*, K02567*, K02568*, K00362, K00363, K03385, K15876), assimilatory nitrate reduction (K00360, K00366, K00367, K00372, K10534, K17877), ammonia assimilation (K00264, K00265, K00266, K01915, K01948), and the extracellular nitrate/nitrite transporter (K02575). Asterisks (*) indicate KOs that are also classified under the denitrification category in the KEGG database, but they were classified as dissimilatory nitrate reduction for all analyses herein. To examine temporal differences in predicted nitrogen cycling gene counts between all nitrogen transformation pathways (see groups of KEGG orthologs above) in May and July 2015, paired two-sided Student’s t-tests were run in R.

### Metagenome sequencing and analysis in MG-RAST

To verify that the nitrogen cycling genes identified in experiment-wide metagenome (PICRUSt) predictions were present in an actual *H. heliophila* metagenome, we constructed a full (sponge and microbial DNA) shotgun sequenced metagenome from DNA extracted from one of the 2014 intertidal *H. heliophila* samples. The same extracted DNA used for 16S rRNA gene sequencing was sent to the University of California Berkeley for shotgun sequencing of ca. 500 bp fragments using Illumina MiSeq standard protocols. Raw sequence output was processed on the MG-RAST server pipeline^63^ (MG-RAST ID 4641054.3), with analyses focused on nitrogen cycling gene content. The KO annotation source^64^ was used to search for KEGG metabolism genes involved in nitrogen cycling, using a maximum e-value of e <10^−5^, a minimum identity of 60%, and a minimum alignment length of 15 (aa for protein and bp for RNA databases). After searching for all genes under the nitrogen metabolism pathway (ko00910), we searched individually for the remaining nitrogen cycling KEGG orthologs that were included in the PICRUSt predictive metagenomic analysis (see above).

### Core microbial community characterization

The core microbial community was defined by identifying microbial OTUs that were present in all 44 *H. heliophila* samples across both years, including 6 intertidal and 6 subtidal sponges from 2014, as well as all sponges from control and experimental treatments in 2015. The absence of ambient seawater samples in the current study did not affect our core OTU definition, as we utilized a membership (i.e. shared presence) definition^65^ commonly employed in sponge microbiome studies^12^,^14^. To determine the putative functionality of the core microbiome, the core metagenome and OTU-specific gene counts for key nitrogen cycling processes (see above) were generated using PICRUSt with a subset of the data that included only the core microbial OTUs (*n* = 18). For the predicted core metagenome, mean NSTI was 0.095 (±0.01).

## Additional Information

**How to cite this article:** Weigel, B. L. and Erwin, P. M. Effects of reciprocal transplantation on the microbiome and putative nitrogen cycling functions of the intertidal sponge, *Hymeniacidon heliophila. Sci. Rep.* **7**, 43247; doi: 10.1038/srep43247 (2017).

**Publisher's note:** Springer Nature remains neutral with regard to jurisdictional claims in published maps and institutional affiliations.

## Supplementary Material

Supplementary Material

## Figures and Tables

**Table 1:** Alpha diversity metrics for microbial communities associated with *H. heliophila* from reciprocal transplant treatments and from pooled treatments in May and July.

Treatment or Month	*S*	*H'*	*D*
Intertidal Control	455 (±17)	3.93 (±0.11)	16.33 (±1.74)
Intertidal to Subtidal	466 (±44)	3.90 (±0.17)	16.74 (±2.52)
Subtidal Control	559 (±52)	4.15 (±0.21)	21.13 (±5.53)
Subtidal to Intertidal	556 (±67)	4.26 (±0.21)	19.68 (±2.96)
May	556 (±33)^a^	4.20 (±0.15)^a^	20.74 (±3.10)^a^
July	441 (±22)^b^	3.84 (±0.07)^b^	15.42 (±1.06)^a^

*S* = OTU richness; *H′* = Shannon index; *D* = inverse Simpson index.

Values denote means (±1SE). No significant differences in diversity metrics were detected among the transplant treatment and control sponges. Significant differences in monthly means are represented by different superscript letters (paired t-tests).

**Table 2:** Pairwise statistical comparisons (PERMANOVA) of *H. heliophila* microbial community structure among the treatment and control sponges in May and July.

Pairwise Comparison	May PERMANOVA		July PERMANOVA	
	*t*	*P*	*t*	*P*
Intertidal Control – Intertidal to Subtidal	0.928	0.614	1.359	0.01*
Intertidal Control – Subtidal Control	1.005	0.393	1.348	0.014*
Intertidal Control – Subtidal to Intertidal	1.242	0.147	1.015	0.503
Subtidal Control – Intertidal to Subtidal	0.790	0.837	1.089	0.215
Subtidal Control – Subtidal to Intertidal	0.975	0.466	1.368	0.058
Subtidal to Intertidal – Intertidal to Subtidal	0.968	0.428	1.305	0.084

Significant pairwise comparisons following Benjamini-Yekutieli corrections are indicated with an asterisk (*).

**Table 3:** Taxonomic classification and average relative abundance (% total community) of core symbiont OTUs (present in all 44 *H. heliophila* samples).

OTU	Phylum	Lowest Taxonomic Classification	Avg. Relative Abundance
1	Proteobacteria	*Novosphingobium resinovorum* (α-proteo.)	17.44
2	Proteobacteria	Order Kiloniellales (α-proteo.)	8.68
3	Proteobacteria	*Thiorhodospira sp.* (γ-proteo.)	6.44
4	Proteobacteria	Family Rhodospirillaceae (α-proteo.)	5.91
5	Planctomycetes	Family Pirellulaceae	6.02
6	Actinobacteria	Order Actinomycetales	3.10
7	Proteobacteria	*Shewanella amazonensis* (γ-proteo.)	3.25
9	Cyanobacteria	*Synechococcus sp.*	2.74
10	Verrucomicrobia	*Persicirhabdus sp.*	1.55
11	Bacteroidetes	Family Flavobacteriaceae	1.61
13	Proteobacteria	*Bartonella bacilliformis* (α-proteo.)	1.24
14	Spirochaetes	*Leptonema sp.*	1.15
15	Cyanobacteria	*Synechococcus sp.*	1.06
16	Proteobacteria	*Bdellovibrio sp.* (*δ*-proteo.)	1.05
17	Planctomycetes	Family Pirellulaceae	0.75
22	Proteobacteria	Family Methylophilaceae (β-proteo.)	0.56
31	Bacteroidetes	Family Flavobacteriaceae	0.38
32	Bacteroidetes	*Flavobacterium sp.*	0.30
