# Supplementary material for: Effects of reciprocal transplantation on the microbiome and putative nitrogen cycling functions of the intertidal sponge, Hymeniacidon heliophila
Source: Sci Rep. 2017 Feb 24;7:43247. doi: 10.1038/srep43247 (PMC5324122; doi:10.1038/srep43247)
Supplement: Supplementary Material [file srep43247-s1.pdf]

SUPPLEMENATRY MATERIAL FOR:

Formatted for: Scientific Reports

Effects of reciprocal transplantation on the microbiome and putative nitrogen cycling functions of the intertidal sponge, *Hymeniacidon heliophila*

Brooke L. Weigel[a#], Patrick M. Erwin[a*]

Department of Biology and Marine Biology, Center for Marine Science, University of North Carolina Wilmington, 5600 Marvin K. Moss Lane, Wilmington, NC, USA 28409[a]

[*]Corresponding Author: Patrick M. Erwin, erwinp@uncw.edu

#Current address: *Committee on Evolutionary Biology, University of Chicago, 1025 E. 57th Street, Chicago, IL 60637*

Table S1. Taxonomy and percentage contribution to community dissimilarity (SIMPER) of microbial OTUs in the sponge *Hymeniacidon heliophila* that exhibited significantly different relative abundances (Metastats, *P* < 0.05) in May and July.

OTU	Metastats p-value	SIMPER	Kingdom	Phylum	Class	Order	Family	Genus	Species
Otu000005	0.000999	4.21	Bacteria	Planctomycetes	Planctomycetia	Pirellulales	Pirellulaceae	unclassified	unclassified
Otu000008	0.000999	7.07	Bacteria	Cyanobacteria	Synechococcophycideae	Synechococcales	Synechococcaceae	Synechococcus	unclassified
Otu000010	0.000999	3.47	Bacteria	Verrucomicrobia	Verrucomicrobiae	Verrucomicrobiales	Verrucomicrobiaceae	unclassified	unclassified
Otu000011	0.016983	1.65	Bacteria	Bacteroidetes	Flavobacteriia	Flavobacteriales	Flavobacteriaceae	unclassified	unclassified
Otu000012	0.001998	0.57	Bacteria	Proteobacteria	Alphaproteobacteria	Rhodobacterales	Rhodobacteraceae	unclassified	unclassified
Otu000017	0.000999	0.91	Bacteria	Planctomycetes	Planctomycetia	Pirellulales	Pirellulaceae	unclassified	unclassified
Otu000018	0.000999	0.57	Bacteria	Proteobacteria	Alphaproteobacteria	Rickettsiales	Pelagibacteraceae	unclassified	unclassified
Otu000019	0.000999	1.34	Bacteria	Proteobacteria	Gammaproteobacteria	Legionellales	Coxiellaceae	Rickettsiella	unclassified
Otu000021	0.000999	0.35	Bacteria	Proteobacteria	Gammaproteobacteria	Oceanospirillales	SUP05	unclassified	unclassified
Otu000022	0.007992	0.47	Bacteria	Proteobacteria	Betaproteobacteria	Methylophilales	Methylophilaceae	unclassified	unclassified
Otu000024	0.000999	0.57	Bacteria	Proteobacteria	unclassified	unclassified	unclassified	unclassified	unclassified
Otu000025	0.000999	1.11	Bacteria	Proteobacteria	Betaproteobacteria	Tremblayales	unclassified	unclassified	unclassified
Otu000026	0.000999	0.45	Bacteria	Proteobacteria	unclassified	unclassified	unclassified	unclassified	unclassified
Otu000027	0.000999	0.11	Bacteria	Actinobacteria	Acidimicrobiia	Acidimicrobiales	OCS155	unclassified	unclassified
Otu000028	0.000999	0.71	Bacteria	Bacteroidetes	Cytophagia	Cytophagales	Amoebophilaceae	SC3-56	unclassified
Otu000029	0.000999	0.71	Bacteria	Proteobacteria	Gammaproteobacteria	Oceanospirillales	HOC21	unclassified	unclassified
Otu000030	0.000999	0.21	Bacteria	Bacteroidetes	Flavobacteriia	Flavobacteriales	Cryomorphaceae	unclassified	unclassified
Otu000031	0.000999	0.4	Bacteria	Bacteroidetes	Flavobacteriia	Flavobacteriales	Flavobacteriaceae	unclassified	unclassified
Otu000033	0.000999	0.61	Bacteria	Actinobacteria	Actinobacteria	Actinomycetales	unclassified	unclassified	unclassified
Otu000034	0.000999	0.63	Bacteria	Bacteroidetes	Flavobacteriia	Flavobacteriales	Flavobacteriaceae	unclassified	unclassified
Otu000036	0.000999	0.15	Bacteria	Proteobacteria	Gammaproteobacteria	unclassified	unclassified	unclassified	unclassified
Otu000037	0.000999	0.44	Bacteria	Bacteroidetes	Flavobacteriia	Flavobacteriales	Flavobacteriaceae	Flavobacterium	unclassified
Otu000038	0.000999	0.43	Bacteria	Proteobacteria	Gammaproteobacteria	unclassified	unclassified	unclassified	unclassified
Otu000040	0.000999	0.18	Bacteria	Proteobacteria	Gammaproteobacteria	Alteromonadales	OM60	unclassified	unclassified
Otu000041	0.000999	0.71	Bacteria	Proteobacteria	Betaproteobacteria	Tremblayales	unclassified	unclassified	unclassified
Otu000042	0.000999	0.61	Bacteria	Proteobacteria	Betaproteobacteria	Tremblayales	unclassified	unclassified	unclassified
Otu000043	0.004995	0.17	Bacteria	Proteobacteria	Alphaproteobacteria	Rhodobacterales	Rhodobacteraceae	unclassified	unclassified
Otu000044	0.000999	0.3	Bacteria	unclassified	unclassified	unclassified	unclassified	unclassified	unclassified
Otu000045	0.024975	0.25	Bacteria	Proteobacteria	Deltaproteobacteria	Bdellovibrionales	Bdellovibrionaceae	Bdellovibrio	unclassified
Otu000048	0.000999	0.17	Bacteria	Proteobacteria	Deltaproteobacteria	Desulfobacterales	Desulfobulbaceae	unclassified	unclassified
Otu000051	0.001998	0.15	Bacteria	Proteobacteria	Deltaproteobacteria	Desulfobacterales	Desulfobulbaceae	unclassified	unclassified
Otu000052	0.000999	0.11	Bacteria	Proteobacteria	Gammaproteobacteria	Oceanospirillales	Halomonadaceae	Candidatus_Portiera	unclassified
Otu000053	0.026973	0.1	Bacteria	Proteobacteria	Alphaproteobacteria	Rickettsiales	Pelagibacteraceae	unclassified	unclassified
Otu000054	0.000999	0.26	Bacteria	Proteobacteria	Betaproteobacteria	Methylophilales	Methylophilaceae	unclassified	unclassified
Otu000059	0.000999	0.23	Bacteria	Proteobacteria	Alphaproteobacteria	Rhodobacterales	Rhodobacteraceae	Phaeobacter	unclassified
Otu000062	0.001998	0.17	Bacteria	Proteobacteria	Gammaproteobacteria	unclassified	unclassified	unclassified	unclassified
Otu000063	0.000999	0.14	Bacteria	Bacteroidetes	Flavobacteriia	Flavobacteriales	Flavobacteriaceae	unclassified	unclassified
Otu000064	0.017982	0.09	Bacteria	Planctomycetes	Planctomycetia	Pirellulales	Pirellulaceae	unclassified	unclassified

Otu000065	0.022977	0.08	Bacteria	Proteobacteria	Gammaproteobacteria	Alteromonadales	OM60	unclassified	unclassified
Otu000068	0.028971	0.08	Bacteria	Proteobacteria	Gammaproteobacteria	Alteromonadales	OM60	Congregibacter	unclassified
Otu000070	0.000999	0.13	Bacteria	Planctomycetes	Planctomycetia	Pirellulales	Pirellulaceae	unclassified	unclassified
Otu000071	0.02997	0.1	Bacteria	Proteobacteria	Gammaproteobacteria	Alteromonadales	HTCC2188	HTCC	unclassified
Otu000072	0.000999	0.12	Bacteria	Actinobacteria	Actinobacteria	Actinomycetales	Microbacteriaceae	Candidatus_Aquiluna	rubra
Otu000073	0.000999	0.18	Bacteria	Bacteroidetes	Cytophagia	Cytophagales	Flammeovirgaceae	unclassified	unclassified
Otu000074	0.000999	0.08	Bacteria	Actinobacteria	Acidimicrobiia	Acidimicrobiales	C111	unclassified	unclassified
Otu000075	0.000999	0.09	Bacteria	Actinobacteria	Actinobacteria	Actinomycetales	Microbacteriaceae	unclassified	unclassified
Otu000080	0.000999	0.17	Bacteria	Proteobacteria	unclassified	unclassified	unclassified	unclassified	unclassified
Otu000082	0.048951	0.14	Bacteria	Proteobacteria	Deltaproteobacteria	Bdellovibrionales	Bdellovibrionaceae	Bdellovibrio	unclassified
Otu000084	0.040959	0.06	Bacteria	Proteobacteria	Gammaproteobacteria	unclassified	unclassified	unclassified	unclassified
Otu000085	0.006993	0.16	Bacteria	Proteobacteria	Alphaproteobacteria	Rhodobacterales	Rhodobacteraceae	unclassified	unclassified
Otu000086	0.000999	0.11	Bacteria	Proteobacteria	Alphaproteobacteria	Rhodobacterales	Rhodobacteraceae	unclassified	unclassified
Otu000087	0.000999	0.16	Bacteria	Proteobacteria	Alphaproteobacteria	Rhodobacterales	Rhodobacteraceae	Marivita	unclassified
Otu000089	0.000999	0.14	Bacteria	Verrucomicrobia	Verrucomicrobiae	Verrucomicrobiales	Verrucomicrobiaceae	Rubritalea	unclassified
Otu000090	0.000999	0.12	Bacteria	Proteobacteria	Alphaproteobacteria	Rhizobiales	Beijerinckiaceae	unclassified	unclassified
Otu000092	0.000999	0.12	Bacteria	Planctomycetes	Planctomycetia	Pirellulales	Pirellulaceae	unclassified	unclassified
Otu000093	0.03996	0.11	Bacteria	Proteobacteria	Alphaproteobacteria	Rhodobacterales	Rhodobacteraceae	Jannaschia	unclassified
Otu000095	0.027972	0.07	Bacteria	Proteobacteria	Gammaproteobacteria	Marinicellales	Marinicellaceae	unclassified	unclassified
Otu000096	0.001998	0.07	Bacteria	Proteobacteria	Gammaproteobacteria	Vibrionales	Vibrionaceae	Photobacterium	damselae
Otu000100	0.000999	0.13	Bacteria	Proteobacteria	unclassified	unclassified	unclassified	unclassified	unclassified
Otu000101	0.000999	0.11	Bacteria	Tenericutes	Mollicutes	Acholeplasmatales	Acholeplasmataceae	Acholeplasma	unclassified
Otu000106	0.000999	0.07	Bacteria	Proteobacteria	Gammaproteobacteria	Thiohalorhabdales	unclassified	unclassified	unclassified
Otu000107	0.040959	0.09	Bacteria	Proteobacteria	Alphaproteobacteria	Rhodobacterales	Rhodobacteraceae	Octadecabacter	antarcticus
Otu000108	0.005994	0.06	Bacteria	Proteobacteria	Gammaproteobacteria	Alteromonadales	OM60	unclassified	unclassified
Otu000110	0.023976	0.06	Bacteria	unclassified	unclassified	unclassified	unclassified	unclassified	unclassified
Otu000111	0.017982	0.09	Bacteria	Planctomycetes	Planctomycetia	Pirellulales	Pirellulaceae	unclassified	unclassified
Otu000112	0.013986	0.06	Bacteria	Proteobacteria	Alphaproteobacteria	Sphingomonadales	Sphingomonadaceae	unclassified	unclassified
Otu000113	0.000999	0.08	Bacteria	Proteobacteria	unclassified	unclassified	unclassified	unclassified	unclassified
Otu000114	0.012987	0.06	Bacteria	Proteobacteria	Alphaproteobacteria	Rhizobiales	Hyphomicrobiaceae	Devosia	unclassified
Otu000115	0.000999	0.11	Bacteria	Proteobacteria	Alphaproteobacteria	unclassified	unclassified	unclassified	unclassified
Otu000116	0.040959	0.28	Bacteria	Proteobacteria	Gammaproteobacteria	Legionellales	Francisellaceae	Francisella	unclassified
Otu000121	0.000999	0.08	Bacteria	Bacteroidetes	Sphingobacteriia	Sphingobacteriales	NS11-12	unclassified	unclassified
Otu000122	0.00999	0.07	Bacteria	Proteobacteria	Alphaproteobacteria	Rhodobacterales	Rhodobacteraceae	Phaeobacter	unclassified
Otu000124	0.000999	0.11	Bacteria	Proteobacteria	unclassified	unclassified	unclassified	unclassified	unclassified
Otu000130	0.000999	0.08	Bacteria	Proteobacteria	Deltaproteobacteria	Bdellovibrionales	Bdellovibrionaceae	Bdellovibrio	unclassified
Otu000131	0.038961	0.04	Bacteria	Proteobacteria	Alphaproteobacteria	Rhodospirillales	Rhodospirillaceae	unclassified	unclassified
Otu000133	0.000999	0.1	Bacteria	Proteobacteria	Betaproteobacteria	Tremblayales	unclassified	unclassified	unclassified
Otu000135	0.018981	0.04	Bacteria	Planctomycetes	Planctomycetia	Planctomycetales	Planctomycetaceae	Planctomyces	unclassified
Otu000136	0.025974	0.07	Bacteria	Proteobacteria	Gammaproteobacteria	Thiotrichales	Piscirickettsiaceae	unclassified	unclassified
Otu000137	0.000518	0.03	Bacteria	Bacteroidetes	Flavobacteriia	Flavobacteriales	Cryomorphaceae	unclassified	unclassified
Otu000138	0.000999	0.06	Bacteria	Proteobacteria	Alphaproteobacteria	Rhodobacterales	Rhodobacteraceae	unclassified	unclassified
Otu000139	0.000999	0.06	Bacteria	Planctomycetes	Planctomycetia	Planctomycetales	Planctomycetaceae	Planctomyces	unclassified
Otu000141	0.004995	0.05	Bacteria	Proteobacteria	Alphaproteobacteria	unclassified	unclassified	unclassified	unclassified
Otu000143	0.015621	0.01	Bacteria	Proteobacteria	Alphaproteobacteria	Rickettsiales	Pelagibacteraceae	unclassified	unclassified
Otu000146	0.001998	0.05	Bacteria	Proteobacteria	Alphaproteobacteria	Rhizobiales	unclassified	unclassified	unclassified

Otu000151	0.015621	0.01	Bacteria	Bacteroidetes	Saprospirae	Saprospirales	Saprospiraceae	Saprospira	unclassified
Otu000153	0.000999	0.05	Bacteria	Cyanobacteria	unclassified	unclassified	unclassified	unclassified	unclassified
Otu000158	0.002997	0.08	Bacteria	Proteobacteria	Deltaproteobacteria	Bdellovibrionales	Bdellovibrionaceae	Bdellovibrio	unclassified
Otu000159	0.013986	0.05	Bacteria	Proteobacteria	Alphaproteobacteria	Rhizobiales	Phyllobacteriaceae	Chelativorans	unclassified
Otu000160	0.000999	0.06	Bacteria	Proteobacteria	unclassified	unclassified	unclassified	unclassified	unclassified
Otu000163	0.039053	0.02	Bacteria	Proteobacteria	Epsilonproteobacteria	Campylobacterales	Helicobacteraceae	Sulfurimonas	unclassified
Otu000164	0.000999	0.04	Bacteria	Cyanobacteria	4C0d-2	YS2	unclassified	unclassified	unclassified
Otu000165	0.000999	0.07	Bacteria	Planctomycetes	Planctomycetia	Pirellulales	Pirellulaceae	unclassified	unclassified
Otu000166	0.000999	0.05	Bacteria	Planctomycetes	Planctomycetia	Planctomycetales	Planctomycetaceae	Planctomyces	unclassified
Otu000174	0.000999	0.07	Bacteria	Proteobacteria	Gammaproteobacteria	Alteromonadales	OM60	unclassified	unclassified
Otu000179	0.000999	0.05	Bacteria	Cyanobacteria	Synechococcophycideae	Synechococcales	Synechococcaceae	Synechococcus	unclassified
Otu000182	0.000244	0.03	Bacteria	Actinobacteria	Acidimicrobiia	Acidimicrobiales	wb1_P06	unclassified	unclassified
Otu000184	0.031245	0.01	Archaea	Euryarchaeota	Thermoplasmata	E2	Marine_group_II	unclassified	unclassified
Otu000185	0.001998	0.04	Bacteria	Verrucomicrobia	Verrucomicrobiae	Verrucomicrobiales	Verrucomicrobiaceae	MSBL3	unclassified
Otu000186	0.000999	0.06	Bacteria	Bacteroidetes	Flavobacteriia	Flavobacteriales	Flavobacteriaceae	unclassified	unclassified
Otu000189	0.000999	0.08	Bacteria	Proteobacteria	Betaproteobacteria	Burkholderiales	Alcaligenaceae	unclassified	unclassified
Otu000191	0.000999	0.05	Bacteria	Proteobacteria	Gammaproteobacteria	Alteromonadales	OM60	unclassified	unclassified
Otu000196	0.022977	0.05	Bacteria	Actinobacteria	Acidimicrobiia	Acidimicrobiales	koll13	unclassified	unclassified
Otu000197	0.022452	0.02	Bacteria	Bacteroidetes	Cytophagia	Cytophagales	Flammeovirgaceae	Roseivirga	unclassified
Otu000198	0.007992	0.04	Bacteria	Planctomycetes	Planctomycetia	Pirellulales	Pirellulaceae	unclassified	unclassified
Otu000200	0.000999	0.04	Bacteria	Proteobacteria	Epsilonproteobacteria	Campylobacterales	Helicobacteraceae	unclassified	unclassified
Otu000203	0.017982	0.05	Bacteria	Acidobacteria	Sva0725	Sva0725	unclassified	unclassified	unclassified
Otu000206	0.002348	0.05	Bacteria	Proteobacteria	Gammaproteobacteria	HOC36	unclassified	unclassified	unclassified
Otu000207	0.000999	0.05	Bacteria	Proteobacteria	Gammaproteobacteria	unclassified	unclassified	unclassified	unclassified
Otu000208	0.036963	0.05	Bacteria	Chlamydiae	Chlamydiia	Chlamydiales	unclassified	unclassified	unclassified
Otu000209	0.000999	0.05	Bacteria	Planctomycetes	Planctomycetia	Pirellulales	Pirellulaceae	unclassified	unclassified
Otu000212	0.000061	0.03	Bacteria	Cyanobacteria	Oscillatoriophycideae	Oscillatoriales	Phormidiaceae	Oscillatoria	spongeliae
Otu000215	0.000488	0.02	Bacteria	Bacteroidetes	Rhodothermi	Rhodothermales	Balneolaceae	Balneola	unclassified
Otu000217	0.000122	0.03	Bacteria	Bacteroidetes	Cytophagia	Cytophagales	Flammeovirgaceae	unclassified	unclassified
Otu000218	0.035142	0.02	Bacteria	Bacteroidetes	Cytophagia	Cytophagales	Flammeovirgaceae	unclassified	unclassified
Otu000220	0.000999	0.04	Bacteria	Proteobacteria	Gammaproteobacteria	Legionellales	Coxiellaceae	Aquicella	unclassified
Otu000221	0.023976	0.04	Bacteria	Bacteroidetes	Flavobacteriia	Flavobacteriales	unclassified	unclassified	unclassified
Otu000232	0.001998	0.03	Bacteria	Proteobacteria	Gammaproteobacteria	Legionellales	Legionellaceae	unclassified	unclassified
Otu000234	0.002997	0.03	Bacteria	Proteobacteria	Alphaproteobacteria	Sphingomonadales	Erythrobacteraceae	Lutibacterium	unclassified
Otu000235	0.000518	0.03	Bacteria	Proteobacteria	Gammaproteobacteria	Oceanospirillales	Halomonadaceae	Candidatus_Portiera	unclassified
Otu000238	0.003996	0.03	Bacteria	Planctomycetes	Planctomycetia	Pirellulales	Pirellulaceae	unclassified	unclassified
Otu000245	0.00183	0.03	Bacteria	Proteobacteria	Gammaproteobacteria	Thiohalorhabdales	unclassified	unclassified	unclassified
Otu000246	0.002348	0.03	Bacteria	Planctomycetes	Planctomycetia	Planctomycetales	Planctomycetaceae	Planctomyces	unclassified
Otu000247	0.030868	0.03	Bacteria	Proteobacteria	Alphaproteobacteria	unclassified	unclassified	unclassified	unclassified
Otu000248	0.000999	0.03	Bacteria	Proteobacteria	Epsilonproteobacteria	Campylobacterales	Helicobacteraceae	unclassified	unclassified
Otu000252	0.000999	0.04	Bacteria	Chlamydiae	Chlamydiia	Chlamydiales	Rhabdochlamydiaceae	Candidatus_Rhabdochlamydia	unclassified
Otu000253	0.000999	0.05	Bacteria	Verrucomicrobia	Opitutae	Puniceicoccales	Puniceicoccaceae	Coraliomargarita	unclassified
Otu000255	0.000488	0.02	Bacteria	Proteobacteria	Deltaproteobacteria	Sva0853	S25_1238	unclassified	unclassified
Otu000258	0.000244	0.03	Bacteria	Bacteroidetes	Flavobacteriia	Flavobacteriales	Cryomorphaceae	Fluviicola	unclassified
Otu000259	0.000999	0.03	Bacteria	Proteobacteria	unclassified	unclassified	unclassified	unclassified	unclassified
Otu000260	0.012718	0.03	Bacteria	Bacteroidetes	Flavobacteriia	Flavobacteriales	Flavobacteriaceae	unclassified	unclassified

Otu000261	0.000999	0.03	Bacteria	Planctomycetes	Planctomycetia	Pirellulales	Pirellulaceae	unclassified	unclassified
Otu000266	0.049021	0.03	Bacteria	Planctomycetes	Planctomycetia	Pirellulales	Pirellulaceae	unclassified	unclassified
Otu000274	0.028971	0.04	Bacteria	Proteobacteria	Gammaproteobacteria	Legionellales	Coxiellaceae	Aquicella	unclassified
Otu000279	0.001952	0.02	Bacteria	Chlamydiae	Chlamydiia	Chlamydiales	unclassified	unclassified	unclassified
Otu000280	0.000488	0.02	Bacteria	Proteobacteria	Betaproteobacteria	Burkholderiales	Comamonadaceae	RS62	unclassified
Otu000285	0.000244	0.03	Bacteria	TM6	SJA-4	unclassified	unclassified	unclassified	unclassified
Otu000286	0.000999	0.03	Bacteria	Proteobacteria	Gammaproteobacteria	Alteromonadales	Alteromonadaceae	Glaciecola	unclassified
Otu000292	0.000976	0.03	Bacteria	Proteobacteria	Alphaproteobacteria	Rhodobacterales	Rhodobacteraceae	Octadecabacter	unclassified
Otu000298	0.000061	0.03	Bacteria	Planctomycetes	Planctomycetia	Gemmatales	Gemmataceae	unclassified	unclassified
Otu000300	0.00183	0.03	Bacteria	Cyanobacteria	Oscillatoriophycideae	Chroococcales	Spirulinaceae	Spirulina	unclassified
Otu000302	0.030868	0.03	Bacteria	Proteobacteria	Deltaproteobacteria	Desulfobacterales	Desulfobulbaceae	Desulfobulbus	unclassified
Otu000308	0.038562	0.02	Bacteria	Bacteroidetes	Cytophagia	Cytophagales	Flammeovirgaceae	unclassified	unclassified
Otu000313	0.006344	0.02	Bacteria	Chlamydiae	Chlamydiia	Chlamydiales	unclassified	unclassified	unclassified
Otu000314	0.012718	0.03	Bacteria	Proteobacteria	Alphaproteobacteria	Rhizobiales	Phyllobacteriaceae	Chelativorans	unclassified
Otu000316	0.000999	0.08	Bacteria	Cyanobacteria	Oscillatoriophycideae	Chroococcales	unclassified	unclassified	unclassified
Otu000324	0.002997	0.04	Bacteria	Verrucomicrobia	Verrucomicrobiae	Verrucomicrobiales	Verrucomicrobiaceae	MSBL3	unclassified
Otu000327	0.015621	0.01	Bacteria	Proteobacteria	Deltaproteobacteria	Myxococcales	Nannocystaceae	Plesiocystis	unclassified
Otu000330	0.001952	0.02	Archaea	unclassified	unclassified	unclassified	unclassified	unclassified	unclassified
Otu000337	0.000976	0.02	Bacteria	Proteobacteria	unclassified	unclassified	unclassified	unclassified	unclassified
Otu000338	0.001952	0.02	Bacteria	Planctomycetes	Planctomycetia	Gemmatales	Isosphaeraceae	unclassified	unclassified
Otu000342	0.012718	0.03	Bacteria	Proteobacteria	Deltaproteobacteria	Desulfobacterales	Desulfobulbaceae	unclassified	unclassified
Otu000344	0.031245	0.01	Bacteria	unclassified	unclassified	unclassified	unclassified	unclassified	unclassified
Otu000345	0.021478	0.02	Bacteria	Planctomycetes	Planctomycetia	Pirellulales	Pirellulaceae	unclassified	unclassified
Otu000347	0.039053	0.02	Bacteria	Planctomycetes	Planctomycetia	Pirellulales	Pirellulaceae	unclassified	unclassified
Otu000348	0.000999	0.03	Bacteria	Proteobacteria	Alphaproteobacteria	Rhodobacterales	Rhodobacteraceae	unclassified	unclassified
Otu000349	0.000976	0.02	Bacteria	unclassified	unclassified	unclassified	unclassified	unclassified	unclassified
Otu000352	0.039053	0.02	Bacteria	Proteobacteria	Deltaproteobacteria	Desulfobacterales	Desulfobulbaceae	Desulforhopalus	unclassified
Otu000358	0.025974	0.03	Bacteria	Cyanobacteria	Oscillatoriophycideae	Chroococcales	Gomphosphaeriaceae	unclassified	unclassified
Otu000360	0.000122	0.03	Bacteria	Proteobacteria	Gammaproteobacteria	Thiotrichales	Piscirickettsiaceae	unclassified	unclassified
Otu000367	0.039053	0.02	Bacteria	Proteobacteria	Alphaproteobacteria	Rhodobacterales	Rhodobacteraceae	unclassified	unclassified
Otu000369	0.039053	0.02	Bacteria	Chlamydiae	Chlamydiia	Chlamydiales	Parachlamydiaceae	unclassified	unclassified
Otu000371	0.038562	0.02	Bacteria	Planctomycetes	Planctomycetia	Planctomycetales	Planctomycetaceae	Planctomyces	unclassified
Otu000372	0.000518	0.03	Bacteria	Proteobacteria	Alphaproteobacteria	Rhodobacterales	Rhodobacteraceae	Loktanella	unclassified
Otu000378	0.031245	0.01	Bacteria	Proteobacteria	Gammaproteobacteria	Alteromonadales	HTCC2188	HTCC	unclassified
Otu000382	0.011714	0.02	Bacteria	Planctomycetes	Planctomycetia	Pirellulales	Pirellulaceae	unclassified	unclassified
Otu000385	0.000976	0.02	Bacteria	Proteobacteria	unclassified	unclassified	unclassified	unclassified	unclassified
Otu000386	0.000244	0.03	Bacteria	Proteobacteria	Betaproteobacteria	Tremblayales	unclassified	unclassified	unclassified
Otu000389	0.039053	0.02	Bacteria	Actinobacteria	Acidimicrobiia	Acidimicrobiales	ZA3409c	unclassified	unclassified
Otu000393	0.003905	0.02	Bacteria	Proteobacteria	Gammaproteobacteria	Legionellales	Coxiellaceae	unclassified	unclassified
Otu000396	0.031245	0.01	Bacteria	Chlamydiae	Chlamydiia	Chlamydiales	unclassified	unclassified	unclassified
Otu000397	0.000976	0.03	Bacteria	Proteobacteria	Alphaproteobacteria	unclassified	unclassified	unclassified	unclassified
Otu000400	0.000976	0.02	Bacteria	Bacteroidetes	Flavobacteriia	Flavobacteriales	Flavobacteriaceae	unclassified	unclassified
Otu000404	0.003905	0.02	Bacteria	Proteobacteria	Gammaproteobacteria	Legionellales	Coxiellaceae	Aquicella	unclassified
Otu000406	0.000244	0.03	Bacteria	Planctomycetes	Planctomycetia	Gemmatales	Isosphaeraceae	unclassified	unclassified
Otu000410	0.000488	0.02	Bacteria	Proteobacteria	unclassified	unclassified	unclassified	unclassified	unclassified
Otu000414	0.00781	0.02	Bacteria	Proteobacteria	Alphaproteobacteria	Rhodobacterales	Rhodobacteraceae	unclassified	unclassified

Otu000415	0.022452	0.02	Bacteria	Proteobacteria	unclassified	unclassified	unclassified	unclassified	unclassified
Otu000416	0.030868	0.03	Bacteria	TM6	SJA-4	unclassified	unclassified	unclassified	unclassified
Otu000419	0.021478	0.02	Bacteria	Planctomycetes	Planctomycetia	Pirellulales	Pirellulaceae	unclassified	unclassified
Otu000424	0.006344	0.02	Bacteria	Proteobacteria	Gammaproteobacteria	Legionellales	Legionellaceae	unclassified	unclassified
Otu000432	0.000488	0.02	Bacteria	Proteobacteria	Alphaproteobacteria	Rhodobacterales	unclassified	unclassified	unclassified
Otu000436	0.015621	0.01	Bacteria	Verrucomicrobia	Verrucomicrobiae	Verrucomicrobiales	Verrucomicrobiaceae	unclassified	unclassified
Otu000440	0.015621	0.01	Bacteria	Proteobacteria	Gammaproteobacteria	Alteromonadales	OM60	unclassified	unclassified
Otu000450	0.000122	0.03	Bacteria	Verrucomicrobia	Verrucomicrobiae	Verrucomicrobiales	Verrucomicrobiaceae	unclassified	unclassified
Otu000458	0.015621	0.01	Bacteria	Proteobacteria	Gammaproteobacteria	unclassified	unclassified	unclassified	unclassified
Otu000466	0.000976	0.02	Bacteria	Planctomycetes	Planctomycetia	Pirellulales	Pirellulaceae	unclassified	unclassified
Otu000470	0.000488	0.02	Bacteria	Cyanobacteria	Synechococcophycideae	Synechococcales	Synechococcaceae	Synechococcus	unclassified
Otu000475	0.000122	0.03	Bacteria	Proteobacteria	Alphaproteobacteria	Rhodospirillales	Rhodospirillaceae	unclassified	unclassified
Otu000478	0.015621	0.01	Bacteria	Verrucomicrobia	Verrucomicrobiae	Verrucomicrobiales	Verrucomicrobiaceae	Luteolibacter	unclassified
Otu000479	0.006344	0.02	Bacteria	Proteobacteria	Gammaproteobacteria	Alteromonadales	Alteromonadaceae	unclassified	unclassified
Otu000480	0.011714	0.02	Bacteria	Bacteroidetes	Flavobacteriia	Flavobacteriales	Cryomorphaceae	Fluviicola	unclassified
Otu000486	0.021478	0.02	Bacteria	Proteobacteria	Deltaproteobacteria	Desulfobacterales	Desulfobacteraceae	Desulfococcus	unclassified
Otu000487	0.003416	0.02	Bacteria	Actinobacteria	Actinobacteria	Actinomycetales	unclassified	unclassified	unclassified
Otu000489	0.000976	0.02	Bacteria	Planctomycetes	Planctomycetia	Pirellulales	Pirellulaceae	unclassified	unclassified
Otu000493	0.015621	0.01	Bacteria	Proteobacteria	Deltaproteobacteria	Desulfobacterales	Desulfobulbaceae	unclassified	unclassified
Otu000494	0.003416	0.02	Bacteria	Proteobacteria	Alphaproteobacteria	Rhodobacterales	Rhodobacteraceae	Amaricoccus	unclassified
Otu000499	0.012933	0.03	Bacteria	WS5	unclassified	unclassified	unclassified	unclassified	unclassified
Otu000500	0.00183	0.03	Bacteria	Proteobacteria	Gammaproteobacteria	Legionellales	Coxiellaceae	unclassified	unclassified
Otu000501	0.006344	0.02	Bacteria	Planctomycetes	C6	d113	unclassified	unclassified	unclassified
Otu000513	0.00781	0.02	Bacteria	Planctomycetes	Planctomycetia	Pirellulales	Pirellulaceae	unclassified	unclassified
Otu000514	0.015621	0.01	Bacteria	Planctomycetes	Planctomycetia	Pirellulales	Pirellulaceae	unclassified	unclassified
Otu000528	0.003905	0.02	Bacteria	Proteobacteria	Gammaproteobacteria	Thiotrichales	Piscirickettsiaceae	unclassified	unclassified
Otu000529	0.031245	0.01	Bacteria	Proteobacteria	Alphaproteobacteria	Rhizobiales	Methylocystaceae	Methylosinus	unclassified
Otu000530	0.003416	0.02	Bacteria	unclassified	unclassified	unclassified	unclassified	unclassified	unclassified
Otu000537	0.015621	0.01	Bacteria	Proteobacteria	Epsilonproteobacteria	Campylobacterales	Helicobacteraceae	unclassified	unclassified
Otu000540	0.00781	0.02	Bacteria	Chlamydiae	Chlamydiia	Chlamydiales	unclassified	unclassified	unclassified
Otu000544	0.003416	0.02	Bacteria	Actinobacteria	Actinobacteria	Actinomycetales	unclassified	unclassified	unclassified
Otu000550	0.003416	0.02	Bacteria	Proteobacteria	Gammaproteobacteria	unclassified	unclassified	unclassified	unclassified
Otu000553	0.011714	0.02	Bacteria	Proteobacteria	Gammaproteobacteria	Alteromonadales	Alteromonadaceae	Candidatus_Endobugula	unclassified
Otu000555	0.000976	0.02	Bacteria	Proteobacteria	Alphaproteobacteria	Sphingomonadales	Erythrobacteraceae	Erythrobacter	unclassified
Otu000568	0.031245	0.01	Bacteria	TM6	SJA-4	unclassified	unclassified	unclassified	unclassified
Otu000574	0.003416	0.03	Bacteria	Proteobacteria	Gammaproteobacteria	Oceanospirillales	Endozoicimonaceae	unclassified	unclassified
Otu000585	0.00781	0.02	Bacteria	Actinobacteria	Actinobacteria	Actinomycetales	unclassified	unclassified	unclassified
Otu000592	0.000122	0.03	Bacteria	Bacteroidetes	Cytophagia	Cytophagales	Flammeovirgaceae	unclassified	unclassified
Otu000594	0.031245	0.01	Bacteria	Bacteroidetes	Flavobacteriia	Flavobacteriales	Flavobacteriaceae	Aquimarina	unclassified
Otu000598	0.039053	0.02	Bacteria	Proteobacteria	Gammaproteobacteria	Pseudomonadales	Pseudomonadaceae	Pseudomonas	veronii
Otu000600	0.000976	0.02	Bacteria	Bacteroidetes	Flavobacteriia	Flavobacteriales	Flavobacteriaceae	unclassified	unclassified
Otu000603	0.000999	0.03	Bacteria	Proteobacteria	Alphaproteobacteria	unclassified	unclassified	unclassified	unclassified
Otu000613	0.011714	0.02	Bacteria	Proteobacteria	Alphaproteobacteria	Kiloniellales	Kiloniellaceae	unclassified	unclassified
Otu000617	0.011714	0.02	Bacteria	unclassified	unclassified	unclassified	unclassified	unclassified	unclassified
Otu000623	0.015621	0.01	Bacteria	Actinobacteria	Acidimicrobiia	Acidimicrobiales	JdFBGBact	unclassified	unclassified
Otu000626	0.011714	0.02	Bacteria	Proteobacteria	Alphaproteobacteria	unclassified	unclassified	unclassified	unclassified

Otu000637	0.022452	0.03	Bacteria	Proteobacteria	Gammaproteobacteria	unclassified	unclassified	unclassified	unclassified
Otu000641	0.003905	0.02	Bacteria	Proteobacteria	Alphaproteobacteria	Rhizobiales	unclassified	unclassified	unclassified
Otu000642	0.015621	0.01	Bacteria	Proteobacteria	Deltaproteobacteria	Desulfobacterales	Desulfobulbaceae	unclassified	unclassified
Otu000645	0.031245	0.01	Bacteria	Planctomycetes	Planctomycetia	Pirellulales	Pirellulaceae	unclassified	unclassified
Otu000650	0.00781	0.02	Bacteria	Proteobacteria	Gammaproteobacteria	Legionellales	Legionellaceae	unclassified	unclassified
Otu000651	0.003905	0.02	Bacteria	Verrucomicrobia	Verrucomicrobiae	Verrucomicrobiales	Verrucomicrobiaceae	Luteolibacter	unclassified
Otu000669	0.00781	0.02	Bacteria	Bacteroidetes	Flavobacteriia	Flavobacteriales	Flavobacteriaceae	Aquimarina	unclassified
Otu000673	0.000488	0.02	Bacteria	Proteobacteria	unclassified	unclassified	unclassified	unclassified	unclassified
Otu000689	0.001952	0	Bacteria	Cyanobacteria	Oscillatoriophycideae	Chroococcales	Xenococcaceae	unclassified	unclassified
Otu000693	0.015621	0.01	Bacteria	Proteobacteria	Alphaproteobacteria	Rhodobacterales	Rhodobacteraceae	unclassified	unclassified
Otu000696	0.015621	0.01	Bacteria	Bacteroidetes	Cytophagia	Cytophagales	Flammeovirgaceae	unclassified	unclassified
Otu000706	0.015621	0	Bacteria	unclassified	unclassified	unclassified	unclassified	unclassified	unclassified
Otu000717	0.015621	0.02	Bacteria	Proteobacteria	Alphaproteobacteria	unclassified	unclassified	unclassified	unclassified
Otu000725	0.031245	0	Bacteria	Proteobacteria	Gammaproteobacteria	unclassified	unclassified	unclassified	unclassified
Otu000727	0.031245	0.01	Bacteria	Verrucomicrobia	Opitutae	Puniceicoccales	Puniceicoccaceae	Coraliomargarita	unclassified
Otu000729	0.039053	0.01	Bacteria	Planctomycetes	OM190	CL500-15	unclassified	unclassified	unclassified
Otu000730	0.031245	0	Bacteria	Proteobacteria	Alphaproteobacteria	Sphingomonadales	Sphingomonadaceae	unclassified	unclassified
Otu000746	0.031245	0.01	Bacteria	Chlamydiae	Chlamydiia	Chlamydiales	unclassified	unclassified	unclassified
Otu000756	0.031245	0.01	Bacteria	Proteobacteria	Gammaproteobacteria	Legionellales	Coxiellaceae	Aquicella	unclassified
Otu000768	0.039053	0.01	Bacteria	Bacteroidetes	unclassified	unclassified	unclassified	unclassified	unclassified
Otu000773	0.031245	0.01	Bacteria	unclassified	unclassified	unclassified	unclassified	unclassified	unclassified
Otu000778	0.001952	0.01	Bacteria	Bacteroidetes	Saprospirae	Saprospirales	Chitinophagaceae	unclassified	unclassified
Otu000791	0.00781	0.01	Bacteria	Proteobacteria	Gammaproteobacteria	unclassified	unclassified	unclassified	unclassified
Otu000806	0.031245	0.01	Bacteria	Proteobacteria	Gammaproteobacteria	Legionellales	Coxiellaceae	Aquicella	unclassified
Otu000808	0.015621	0.02	Bacteria	Planctomycetes	Planctomycetia	Pirellulales	Pirellulaceae	unclassified	unclassified
Otu000809	0.001952	0.01	Bacteria	WS5	unclassified	unclassified	unclassified	unclassified	unclassified
Otu000815	0.015621	0.01	Bacteria	Proteobacteria	Gammaproteobacteria	unclassified	unclassified	unclassified	unclassified
Otu000818	0.015621	0	Bacteria	Proteobacteria	Gammaproteobacteria	unclassified	unclassified	unclassified	unclassified
Otu000823	0.00781	0.01	Bacteria	Proteobacteria	Alphaproteobacteria	Rhizobiales	Cohaesibacteraceae	unclassified	unclassified
Otu000825	0.039053	0.01	Bacteria	unclassified	unclassified	unclassified	unclassified	unclassified	unclassified
Otu000841	0.031245	0.01	Bacteria	Proteobacteria	Alphaproteobacteria	unclassified	unclassified	unclassified	unclassified
Otu000853	0.00781	0.01	Bacteria	Proteobacteria	Gammaproteobacteria	unclassified	unclassified	unclassified	unclassified
Otu000856	0.003905	0.01	Bacteria	Planctomycetes	Planctomycetia	Pirellulales	Pirellulaceae	unclassified	unclassified
Otu000858	0.031245	0.01	Bacteria	Planctomycetes	Planctomycetia	Pirellulales	Pirellulaceae	unclassified	unclassified
Otu000865	0.00781	0.01	Bacteria	Bacteroidetes	Flavobacteriia	Flavobacteriales	Flavobacteriaceae	unclassified	unclassified
Otu000877	0.015621	0.01	Bacteria	unclassified	unclassified	unclassified	unclassified	unclassified	unclassified
Otu000882	0.039053	0.01	Bacteria	Planctomycetes	Planctomycetia	Pirellulales	Pirellulaceae	unclassified	unclassified
Otu000883	0.015621	0.01	Bacteria	Proteobacteria	Gammaproteobacteria	Vibrionales	Vibrionaceae	Vibrio	unclassified
Otu000884	0.031245	0.01	Bacteria	Proteobacteria	Alphaproteobacteria	unclassified	unclassified	unclassified	unclassified
Otu000914	0.031245	0.01	Bacteria	Proteobacteria	Alphaproteobacteria	Rhodobacterales	Rhodobacteraceae	unclassified	unclassified
Otu000949	0.00781	0.02	Bacteria	Bacteroidetes	Flavobacteriia	Flavobacteriales	Cryomorphaceae	unclassified	unclassified
Otu000969	0.015621	0.01	Bacteria	Bacteroidetes	Cytophagia	Cytophagales	Cyclobacteriaceae	unclassified	unclassified
Otu001061	0.015621	0.01	Bacteria	WS5	unclassified	unclassified	unclassified	unclassified	unclassified
Otu001081	0.031245	0.01	Bacteria	Verrucomicrobia	Verrucomicrobiae	Verrucomicrobiales	Verrucomicrobiaceae	unclassified	unclassified
Otu001112	0.031245	0.01	Bacteria	Planctomycetes	Planctomycetia	B97	unclassified	unclassified	unclassified
Otu001150	0.00781	0.02	Bacteria	Planctomycetes	Planctomycetia	Planctomycetales	Planctomycetaceae	Planctomyces	unclassified

Otu001191	0.015621	0.01	Bacteria	Proteobacteria	Alphaproteobacteria	Rhodobacterales	Rhodobacteraceae	Pseudoruegeria	unclassified
Otu001242	0.031245	0.01	Bacteria	Proteobacteria	Alphaproteobacteria	Rhodobacterales	Rhodobacteraceae	unclassified	unclassified
Otu001266	0.015621	0.01	Bacteria	Bacteroidetes	Cytophagia	Cytophagales	Flammeovirgaceae	unclassified	unclassified
Otu001287	0.015621	0.01	Bacteria	Bacteroidetes	Flavobacteriia	Flavobacteriales	Flavobacteriaceae	Polaribacter	unclassified
Otu001294	0.011714	0.02	Bacteria	Cyanobacteria	unclassified	unclassified	unclassified	unclassified	unclassified
Otu001312	0.031245	0.01	Bacteria	TM6	SJA-4	unclassified	unclassified	unclassified	unclassified
Otu001342	0.000976	0.02	Bacteria	Proteobacteria	Alphaproteobacteria	Rhodospirillales	Rhodospirillaceae	Rhodovibrio	unclassified
Otu001381	0.031245	0.01	Bacteria	Planctomycetes	Planctomycetia	Pirellulales	Pirellulaceae	unclassified	unclassified
Otu001517	0.031245	0.01	Bacteria	Proteobacteria	Gammaproteobacteria	Legionellales	Coxiellaceae	unclassified	unclassified
Otu001882	0.031245	0.01	Bacteria	Cyanobacteria	Oscillatoriophycideae	unclassified	unclassified	unclassified	unclassified
Otu001888	0.031245	0.01	Bacteria	Chloroflexi	Anaerolineae	Caldilineales	Caldilineaceae	unclassified	unclassified
Otu001907	0.031245	0.01	Bacteria	Chlamydiae	Chlamydiia	Chlamydiales	Parachlamydiaceae	unclassified	unclassified
Otu002135	0.031245	0.01	Bacteria	Verrucomicrobia	Spartobacteria	Chthoniobacterales	Chthoniobacteraceae	Candidatus_Xiphinematobacter	unclassified
Otu003109	0.015621	0.01	Bacteria	Bacteroidetes	Flavobacteriia	Flavobacteriales	Flavobacteriaceae	Arenibacter	unclassified

Table S2. Taxonomy and percentage contribution to community dissimilarity (SIMPER) of microbial OTUs in the sponge *Hymeniacidon heliophila* that exhibited significantly different relative abundances (Metastats, $P < 0.05$) among treatments (IC = intertidal control, SC = subtidal control, IS = intertidal-to-subtidal transplant).

Treatment	OTU	Metastats p-value	SIMPER	Kingdom	Phylum	Class	Order	Family	Genus	Species
IC vs. SC	Otu000001	0.006049	11.12	Bacteria	Proteobacteria	Alphaproteobacteria	Sphingomonadales	Sphingomonadaceae	unclassified	unclassified
IC vs. SC	Otu000010	0.001721	0.19	Bacteria	Verrucomicrobia	Verrucomicrobiae	Verrucomicrobiales	Verrucomicrobiaceae	unclassified	unclassified
IC vs. SC	Otu000012	0.014633	1.23	Bacteria	Proteobacteria	Alphaproteobacteria	Rhodobacterales	Rhodobacteraceae	unclassified	unclassified
IC vs. SC	Otu000013	0.024929	0.66	Bacteria	Proteobacteria	Alphaproteobacteria	unclassified	unclassified	unclassified	unclassified
IC vs. SC	Otu000019	0.005204	0.27	Bacteria	Proteobacteria	Gammaproteobacteria	Legionellales	Coxiellaceae	Rickettsiella	unclassified
IC vs. SC	Otu000034	0.034279	0.45	Bacteria	Bacteroidetes	Flavobacteriia	Flavobacteriales	Flavobacteriaceae	unclassified	unclassified
IC vs. SC	Otu000037	0.005305	0.1	Bacteria	Bacteroidetes	Flavobacteriia	Flavobacteriales	Flavobacteriaceae	Flavobacterium	unclassified
IC vs. SC	Otu000066	0.012566	0.2	Bacteria	Bacteroidetes	Flavobacteriia	Flavobacteriales	Flavobacteriaceae	Lutimonas	unclassified
IC vs. SC	Otu000070	0.00008	0.21	Bacteria	Planctomycetes	Planctomycetia	Pirellulales	Pirellulaceae	unclassified	unclassified
IC vs. SC	Otu000081	0.018336	0.08	Bacteria	Proteobacteria	Gammaproteobacteria	Alteromonadales	OM60	unclassified	unclassified
IC vs. SC	Otu000083	0.021416	0.08	Bacteria	Proteobacteria	Deltaproteobacteria	Desulfobacterales	Desulfobacteraceae	Desulfococcus	unclassified
IC vs. SC	Otu000084	0.049796	0.04	Bacteria	Proteobacteria	Gammaproteobacteria	unclassified	unclassified	unclassified	unclassified
IC vs. SC	Otu000112	0.01854	0.05	Bacteria	Proteobacteria	Alphaproteobacteria	Sphingomonadales	Sphingomonadaceae	unclassified	unclassified
IC vs. SC	Otu000144	0.008173	0.08	Bacteria	Proteobacteria	Gammaproteobacteria	unclassified	unclassified	unclassified	unclassified
IC vs. SC	Otu000181	0.034916	0.08	Bacteria	unclassified	unclassified	unclassified	unclassified	unclassified	unclassified
IC vs. SC	Otu000201	0.009571	0.04	Bacteria	Proteobacteria	Gammaproteobacteria	unclassified	unclassified	unclassified	unclassified
IC vs. SC	Otu000224	0.040575	0.04	Bacteria	Chlamydiae	Chlamydiia	Chlamydiales	Rhabdochlamydiaceae	Candidatus_Rhabdochlamydia	unclassified
IC vs. SC	Otu000236	0.017624	0.07	Bacteria	Planctomycetes	Phycisphaerae	Phycisphaerales	unclassified	unclassified	unclassified
IC vs. SC	Otu000276	0.040575	0.04	Bacteria	Actinobacteria	Acidimicrobiia	Acidimicrobiales	C111	unclassified	unclassified
IC vs. SC	Otu000354	0.049796	0.04	Bacteria	Proteobacteria	Gammaproteobacteria	Alteromonadales	OM60	unclassified	unclassified
IC vs. SC	Otu000358	0.033823	0.11	Bacteria	Cyanobacteria	Oscillatoriophycideae	Chroococcales	Gomphosphaeriaceae	unclassified	unclassified
IC vs. SC	Otu000428	0.008788	0.05	Bacteria	Proteobacteria	Alphaproteobacteria	Rhizobiales	Hyphomicrobiaceae	unclassified	unclassified
IC vs. SC	Otu000530	0.032876	0.06	Bacteria	unclassified	unclassified	unclassified	unclassified	unclassified	unclassified
IC vs. SC	Otu000622	0.040575	0.04	Bacteria	Actinobacteria	Acidimicrobiia	Acidimicrobiales	koll13	unclassified	unclassified
IC vs. SC	Otu000808	0.040575	0	Bacteria	Planctomycetes	Planctomycetia	Pirellulales	Pirellulaceae	unclassified	unclassified
IC vs. SC	Otu000926	0.022765	0.05	Bacteria	Proteobacteria	Gammaproteobacteria	unclassified	unclassified	unclassified	unclassified
IC vs. IS	Otu000001	0.009887	8.29	Bacteria	Proteobacteria	Alphaproteobacteria	Sphingomonadales	Sphingomonadaceae	unclassified	unclassified
IC vs. IS	Otu000010	0.040657	0.11	Bacteria	Verrucomicrobia	Verrucomicrobiae	Verrucomicrobiales	Verrucomicrobiaceae	unclassified	unclassified
IC vs. IS	Otu000012	0.007279	1.26	Bacteria	Proteobacteria	Alphaproteobacteria	Rhodobacterales	Rhodobacteraceae	unclassified	unclassified
IC vs. IS	Otu000013	0.023167	0.84	Bacteria	Proteobacteria	Alphaproteobacteria	unclassified	unclassified	unclassified	unclassified
IC vs. IS	Otu000019	0.008696	0.26	Bacteria	Proteobacteria	Gammaproteobacteria	Legionellales	Coxiellaceae	Rickettsiella	unclassified
IC vs. IS	Otu000022	0.004064	0.5	Bacteria	Proteobacteria	Betaproteobacteria	Methylophilales	Methylophilaceae	unclassified	unclassified
IC vs. IS	Otu000040	0.041569	0.33	Bacteria	Proteobacteria	Gammaproteobacteria	Alteromonadales	OM60	unclassified	unclassified
IC vs. IS	Otu000043	0.017897	0.21	Bacteria	Proteobacteria	Alphaproteobacteria	Rhodobacterales	Rhodobacteraceae	unclassified	unclassified
IC vs. IS	Otu000046	0.042358	0.21	Bacteria	Proteobacteria	Alphaproteobacteria	Rhodobacterales	Rhodobacteraceae	unclassified	unclassified
IC vs. IS	Otu000050	0.048647	0.15	Bacteria	Proteobacteria	Gammaproteobacteria	Marinicellales	Marinicellaceae	unclassified	unclassified
IC vs. IS	Otu000052	0.009113	0.16	Bacteria	Proteobacteria	Gammaproteobacteria	Oceanospirillales	Halomonadaceae	Candidatus_Portiera	unclassified
IC vs. IS	Otu000064	0.001863	0.18	Bacteria	Planctomycetes	Planctomycetia	Pirellulales	Pirellulaceae	unclassified	unclassified
IC vs. IS	Otu000066	0.002564	0.25	Bacteria	Bacteroidetes	Flavobacteriia	Flavobacteriales	Flavobacteriaceae	Lutimonas	unclassified
IC vs. IS	Otu000067	0.027005	0.15	Bacteria	Proteobacteria	Gammaproteobacteria	unclassified	unclassified	unclassified	unclassified
IC vs. IS	Otu000083	0.001417	0.1	Bacteria	Proteobacteria	Deltaproteobacteria	Desulfobacterales	Desulfobacteraceae	Desulfococcus	unclassified

IC vs. IS	Otu000085	0.00799	0.06	Bacteria	Proteobacteria	Alphaproteobacteria	Rhodobacterales	Rhodobacteraceae	unclassified	unclassified
IC vs. IS	Otu000086	0.034299	0.04	Bacteria	Proteobacteria	Alphaproteobacteria	Rhodobacterales	Rhodobacteraceae	unclassified	unclassified
IC vs. IS	Otu000102	0.006152	0.05	Bacteria	Proteobacteria	Gammaproteobacteria	Oceanospirillales	Alcanivoracaceae	Alcanivorax	unclassified
IC vs. IS	Otu000107	0.043691	0.04	Bacteria	Proteobacteria	Alphaproteobacteria	Rhodobacterales	Rhodobacteraceae	Octadecabacter	antarcticus
IC vs. IS	Otu000132	0.043691	0.04	Bacteria	Proteobacteria	Deltaproteobacteria	Desulfobacterales	Desulfobacteraceae	Desulfosarcina	unclassified
IC vs. IS	Otu000144	0.021412	0.07	Bacteria	Proteobacteria	Gammaproteobacteria	unclassified	unclassified	unclassified	unclassified
IC vs. IS	Otu000148	0.009505	0.08	Bacteria	Chlamydiae	Chlamydiia	Chlamydiales	Simkaniaceae	unclassified	unclassified
IC vs. IS	Otu000150	0.046711	0.04	Bacteria	Proteobacteria	Alphaproteobacteria	Rhizobiales	unclassified	unclassified	unclassified
IC vs. IS	Otu000157	0.024225	0.08	Bacteria	Proteobacteria	Gammaproteobacteria	unclassified	unclassified	unclassified	unclassified
IC vs. IS	Otu000177	0.034299	0.04	Bacteria	Proteobacteria	Gammaproteobacteria	unclassified	unclassified	unclassified	unclassified
IC vs. IS	Otu000183	0.043691	0.04	Bacteria	Proteobacteria	Gammaproteobacteria	unclassified	unclassified	unclassified	unclassified
IC vs. IS	Otu000187	0.030593	0.06	Bacteria	Proteobacteria	Gammaproteobacteria	HTCC2188	HTCC2089	unclassified	unclassified
IC vs. IS	Otu000201	0.00749	0.04	Bacteria	Proteobacteria	Gammaproteobacteria	unclassified	unclassified	unclassified	unclassified
IC vs. IS	Otu000204	0.017186	0.07	Bacteria	Planctomycetes	Planctomycetia	Pirellulales	Pirellulaceae	unclassified	unclassified
IC vs. IS	Otu000213	0.018088	0.07	Bacteria	Proteobacteria	Gammaproteobacteria	unclassified	unclassified	unclassified	unclassified
IC vs. IS	Otu000225	0.027451	0.07	Bacteria	Actinobacteria	Acidimicrobiia	Acidimicrobiales	C111	unclassified	unclassified
IC vs. IS	Otu000242	0.034299	0.04	Bacteria	Proteobacteria	Alphaproteobacteria	unclassified	unclassified	unclassified	unclassified
IC vs. IS	Otu000258	0.046711	0.04	Bacteria	Bacteroidetes	Flavobacteriia	Flavobacteriales	Cryomorphaceae	Fluviicola	unclassified
IC vs. IS	Otu000267	0.034299	0.04	Bacteria	Proteobacteria	Gammaproteobacteria	unclassified	unclassified	unclassified	unclassified
IC vs. IS	Otu000276	0.034299	0.04	Bacteria	Actinobacteria	Acidimicrobiia	Acidimicrobiales	C111	unclassified	unclassified
IC vs. IS	Otu000354	0.029265	0.05	Bacteria	Proteobacteria	Gammaproteobacteria	Alteromonadales	OM60	unclassified	unclassified
IC vs. IS	Otu000358	0.017578	0.13	Bacteria	Cyanobacteria	Oscillatoriophycideae	Chroococcales	Gomphosphaeriaceae	unclassified	unclassified
IC vs. IS	Otu000407	0.043691	0.04	Bacteria	Chloroflexi	Anaerolineae	GCA004	unclassified	unclassified	unclassified
IC vs. IS	Otu000428	0.028299	0.05	Bacteria	Proteobacteria	Alphaproteobacteria	Rhizobiales	Hyphomicrobiaceae	unclassified	unclassified
IC vs. IS	Otu000528	0.013922	0.06	Bacteria	Proteobacteria	Gammaproteobacteria	Thiotrichales	Piscirickettsiaceae	unclassified	unclassified
IC vs. IS	Otu000530	0.019608	0.06	Bacteria	unclassified	unclassified	unclassified	unclassified	unclassified	unclassified
IC vs. IS	Otu000622	0.034299	0.04	Bacteria	Actinobacteria	Acidimicrobiia	Acidimicrobiales	koll13	unclassified	unclassified

Table S3. Nitrogen metabolism KEGG orthologs (KOs) identified in the predicted metagenome (PICRUSt) and the shotgun-sequenced metagenome, including overlapping KOs, from one intertidal *H. heliophila* sample.

KEGG Ortholog	Data Source	Function	Category
K00265	both	glutamate synthase (NADPH/NADH) large chain (gltB)	Ammonia assimilation
K00266	both	glutamate synthase (gltD)	Ammonia assimilation
K01915	PICRUSt	glutamine synthetase (glnA)	Ammonia assimilation
K00459	shotgun	nitronate monooxygenase (ncd2, npd)	Ammonia oxidation
K05601	both	hydroxylamine reductase (hcp)	Ammonia oxidation
10535	both	hydroxylamine dehydrogenase (hao)	Ammonia oxidation
00360	shotgun	nitrate reductase (NADH)	Assimilatory nitrate reduction
K00366	both	ferredoxin-nitrite reductase (nirA)	Assimilatory nitrate reduction
K00367	both	ferredoxin-nitrate reductase (narB)	Assimilatory nitrate reduction
K00372	both	assimilatory nitrate reductase catalytic subunit (nasA)	Assimilatory nitrate reduction
K08345	shotgun	nitrate reductase alpha subunit (narZ)	Assimilatory nitrate reduction
K00368	both	nitrite reductase (nirK)	Denitrification
K00376	both	nitrous oxide reductase (nosZ)	Denitrification
K02305	PICRUSt	nitric oxide reductase subunit C (norC)	Denitrification
K02448	shotgun	nitric-oxide reductase, cytochrome c-containing subunit II (norC)	Denitrification
K04561	both	nitric oxide reductase subunit B (norB)	Denitrification
K04748	shotgun	nitric-oxide reductase (norF)	Denitrification
K00362	both	nitrite reductase large subunit (nirB)	Dissimilatory nitrate reduction
K00363	PICRUSt	nitrite reductase (nirD)	Dissimilatory nitrate reduction
K00370	PICRUSt	nitrate reductase alpha subunit (narG)	Dissimilatory nitrate reduction
K00371	PICRUSt	nitrate reductase beta subunit (narH)	Dissimilatory nitrate reduction
K00373	PICRUSt	nitrate reductase delta subunit (narJ)	Dissimilatory nitrate reduction
K00374	PICRUSt	nitrate reductase gamma subunit (narI)	Dissimilatory nitrate reduction
K02567	both	periplasmic nitrate reductase (NapA)	Dissimilatory nitrate reduction
K02568	PICRUSt	cytochrome c-type protein (NapB)	Dissimilatory nitrate reduction
K02569	shotgun	cytochrome c-type protein (napC)	Dissimilatory nitrate reduction
K03385	PICRUSt	nitrite reductase (cytochrome c-552) (nrfA)	Dissimilatory nitrate reduction
K04016	shotgun	formate-dependent nitrate reductase, transmembrane protein (nrfD)	Dissimilatory nitrate reduction
K02575	PICRUSt	extracellular nitrate/nitrite transporter (NRT)	Nitrate transporter
K02586	PICRUSt	nitrogenase molybdenum-iron protein alpha chain (nifD)	Nitrogen fixation
K02588	PICRUSt	nitrogenase iron protein (nifH)	Nitrogen fixation
K02591	PICRUSt	nitrogenase molybdenum-iron protein beta chain (nifK)	Nitrogen fixation
K00369	shotgun	carbonic anhydrase (cah)	Carbonic anhydrase
K01672	shotgun	carbonic anhydrase	Carbonic anhydrase
K01673	shotgun	carbonic anhydrase (cynT, can)	Carbonic anhydrase

Table S4. Multiplex identifier (MID) sequence barcodes and raw read counts corresponding to samples in this study. Samples from the 2015 reciprocal transplant experiment were submitted to the NCBI Sequence Read Archive under the accession number SRP076523, and samples from 2014 intertidal and subtidal sponges were previously submitted under the accession number SRP065064.

MID Sequence	Sample	Source	Raw Read Count
GAGAGTGT	IC2A	Intertidal-Control Sponge (May 2015)	21450
GAGATCAG	IC2B	Intertidal-Control Sponge (July 2015)	19931
GAGATCTC	IC3A	Intertidal-Control Sponge (May 2015)	25411
GAGATGAC	IC3B	Intertidal-Control Sponge (July 2015)	5715
GAGATGTG	IC5A	Intertidal-Control Sponge (May 2015)	19910
GAGTACAG	IC5B	Intertidal-Control Sponge (July 2015)	11752
GAGTACTC	IC6A	Intertidal-Control Sponge (May 2015)	24360
GAGTAGAC	IC6B	Intertidal-Control Sponge (July 2015)	14525
GAGTAGTG	IC8A	Intertidal-Control Sponge (May 2015)	16498
GAGTCACT	IC8B	Intertidal-Control Sponge (July 2015)	16094
GAGTCAGA	IS1A	Intertidal-to-Subtidal Sponge (May 2015)	22285
GAGTCTCA	IS1B	Intertidal-to-Subtidal Sponge (July 2015)	29498
GAGTCTGT	IS2A	Intertidal-to-Subtidal Sponge (May 2015)	21076
GAGTGACA	IS2B	Intertidal-to-Subtidal Sponge (July 2015)	7436
GAGTGAGT	IS4A	Intertidal-to-Subtidal Sponge (May 2015)	21813
GAGTGTCT	IS4B	Intertidal-to-Subtidal Sponge (July 2015)	7939
GAGTGTGA	IS6A	Intertidal-to-Subtidal Sponge (May 2015)	23623
GAGTTCAC	IS6B	Intertidal-to-Subtidal Sponge (July 2015)	37143
GAGTTCTG	IS8A	Intertidal-to-Subtidal Sponge (May 2015)	20459
GAGTTGAG	IS8B	Intertidal-to-Subtidal Sponge (July 2015)	28591
GAGTTGTC	SC2A	Subtidal-Control Sponge (May 2015)	27293
GATCACCA	SC2B	Subtidal-Control Sponge (July 2015)	24187
GATCACGT	SC5A	Subtidal-Control Sponge (May 2015)	32456
GATCAGCT	SC5B	Subtidal-Control Sponge (July 2015)	10344
GATCAGGA	SC6A	Subtidal-Control Sponge (May 2015)	13879
GATCCAAC	SC6B	Subtidal-Control Sponge (July 2015)	52410
GATCCATG	SC8A	Subtidal-Control Sponge (May 2015)	11568
GATCCTAG	SC8B	Subtidal-Control Sponge (July 2015)	32148
GATCCTTC	SI1A	Subtidal-to-Intertidal Sponge (May 2015)	53831
GATCGAAG	SI1B	Subtidal-to-Intertidal Sponge (July 2015)	27625
GATCGATC	SI6A	Subtidal-to-Intertidal Sponge (May 2015)	50688
GATCGTAC	SI6B	Subtidal-to-Intertidal Sponge (July 2015)	4693

GAGTACTC	I1	Intertidal Sponge (July 2014)	12488
GAGTAGAC	I2	Intertidal Sponge (July 2014)	8734
GAGTAGTG	I3	Intertidal Sponge (July 2014)	12289
GAGTCACT	I4	Intertidal Sponge (July 2014)	11977
GAGTCAGA	I5	Intertidal Sponge (July 2014)	15218
GAGTCTCA	I6	Intertidal Sponge (July 2014)	12484
GAGAGTGT	S1	Subtidal Sponge (July 2014)	9994
GAGATCAG	S2	Subtidal Sponge (July 2014)	7533
GAGATCTC	S3	Subtidal Sponge (July 2014)	13390
GAGATGAC	S4	Subtidal Sponge (July 2014)	2853
GAGATGTG	S5	Subtidal Sponge (July 2014)	4148
GAGTACAG	S6	Subtidal Sponge (July 2014)	10556

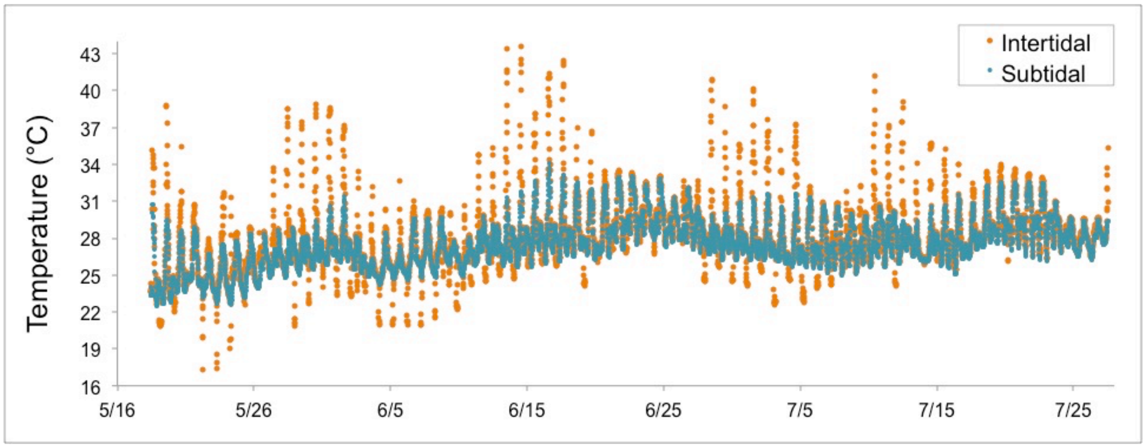

Figure S1. Temperatures from intertidal (orange) and subtidal (blue) control sponges recorded at 15-minute intervals over the 70 days of reciprocal transplantation, from 18 May 2015 to 27 July 2015. Intertidal sponges experienced greater daily temperature fluctuations and extremes than subtidal sponges, despite similar average temperatures in the two environments.

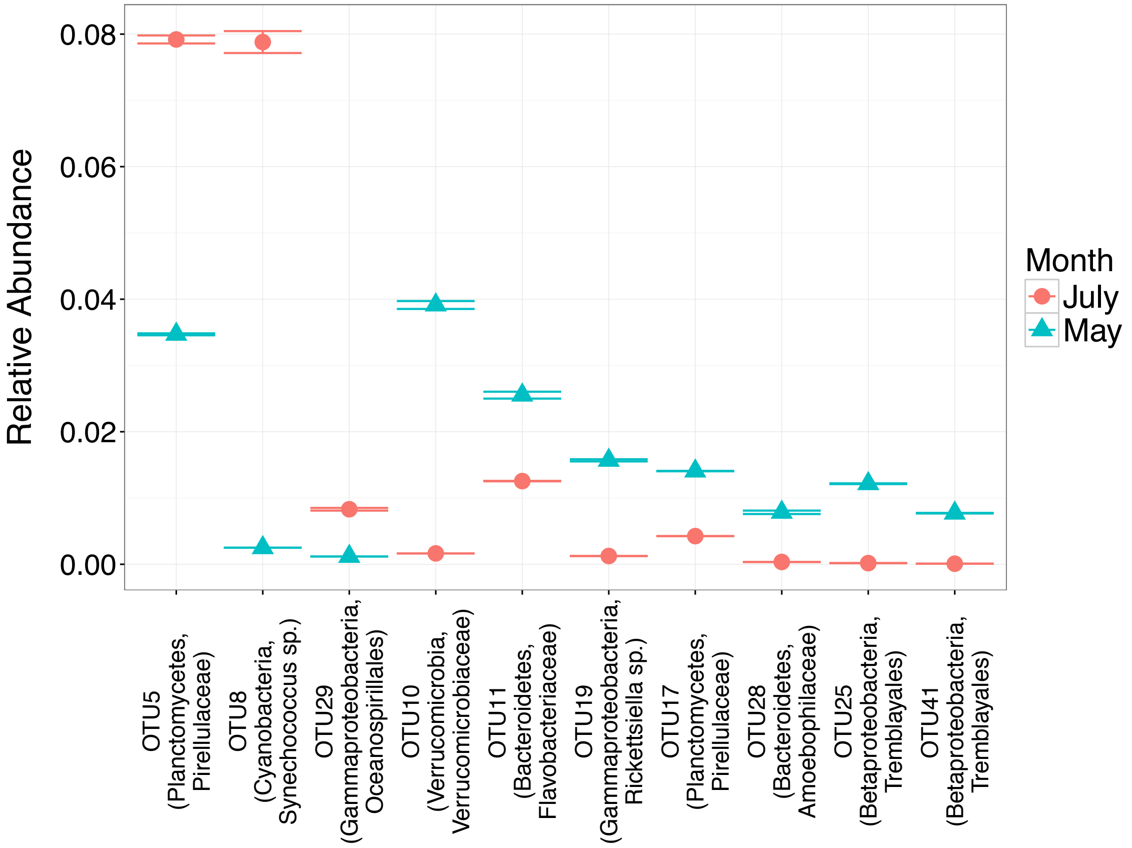

Figure S2. Relative abundances of the top ten OTUs that contributed to the differences in microbial community composition between May and July 2015. These OTUs contributed to a cumulative 22% of the dissimilarity between May and July (as revealed by SIMPER) and had significantly different relative abundances (Metastats, $P < 0.001$).

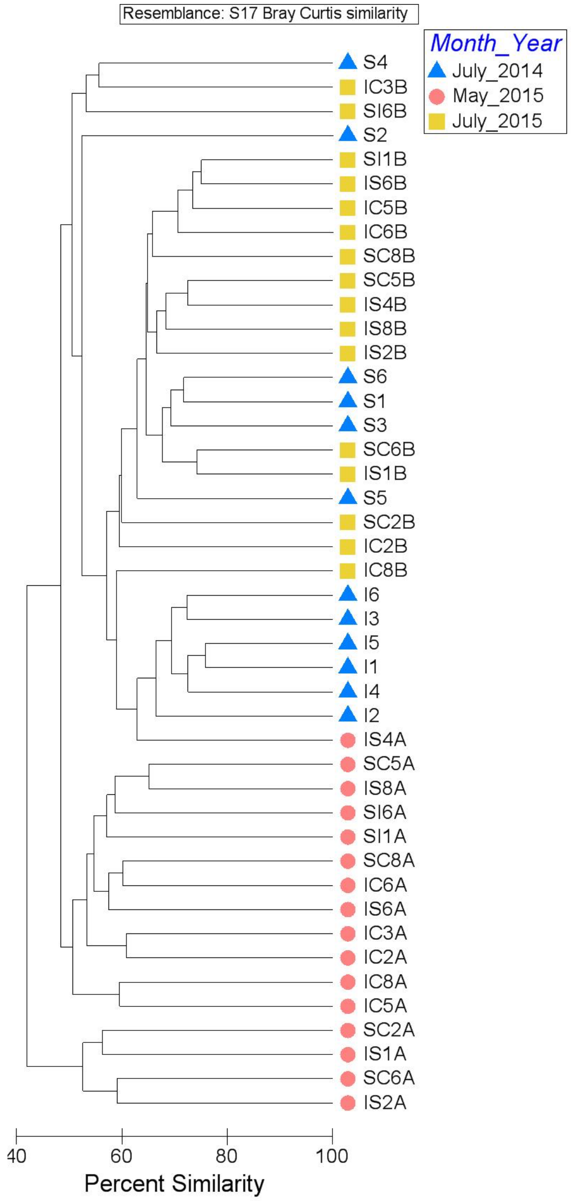

Figure S3. Cluster dendrogram displaying microbial symbiont community similarity among *H. heliophila* from May 2015 (pink circles), July 2015 (yellow squares), and July 2014 (blue triangles). July 2014 samples collected from intertidal (I) and subtidal (S) environments exhibited a high degree of similarity to July 2015 samples. Interestingly, July 2014 intertidal sponges formed a distinct cluster within the July samples.

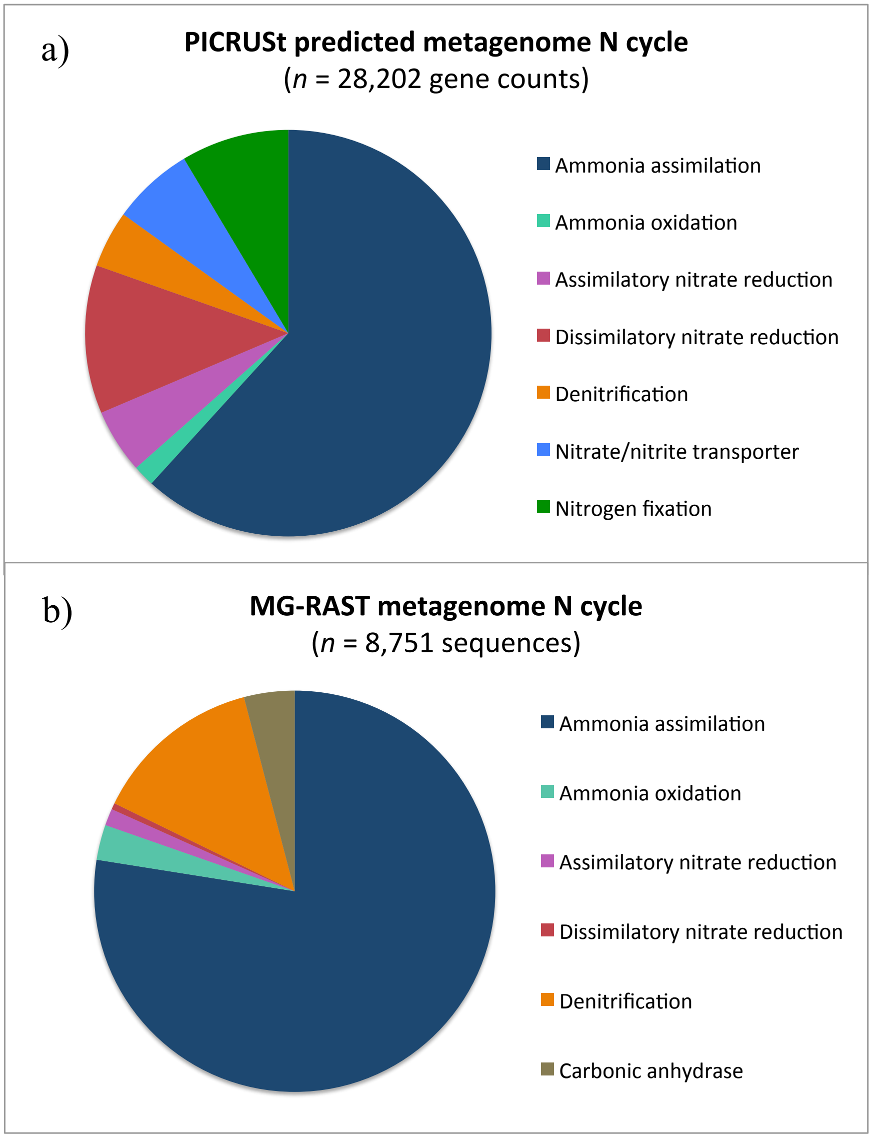

Figure S4. Nitrogen transformation pathways in the *H. heliophila* holobiont from a) the PICRUSt predicted metagenome and b) the MG-RAST shotgun sequenced metagenome of one intertidal sponge sample.
